# SCAF4 and SCAF8, mRNA Anti-Terminator Proteins

**DOI:** 10.1016/j.cell.2019.04.038

**Published:** 2019-06-13

**Authors:** Lea H. Gregersen, Richard Mitter, Alejandro P. Ugalde, Takayuki Nojima, Nicholas J. Proudfoot, Reuven Agami, Aengus Stewart, Jesper Q. Svejstrup

**Affiliations:** 1 Mechanisms of Transcription Laboratory, The Francis Crick Institute, 1 Midland Road, London NW1 1AT, UK; 2 Bioinformatics and Biostatistics, The Francis Crick Institute, 1 Midland Road, London NW1 1AT, UK; 3 The Netherlands Cancer Institute (NKI-AVL), Plesmanlaan 121, 1066 CX Amsterdam, Netherlands; 4 Sir William Dunn School of Pathology, University of Oxford, South Parks Road, OX1 3RE Oxford, UK

**Keywords:** SCAF4, SCAF8, RNA polymerase II, C-terminal repeat domain (CTD), CTD phosphorylation, transcriptional termination, transcript elongation, co-transcriptional mRNA processing, polyadenylation, anti-termination

## Abstract

Accurate regulation of mRNA termination is required for correct gene expression. Here, we describe a role for SCAF4 and SCAF8 as anti-terminators, suppressing the use of early, alternative polyadenylation (polyA) sites. The SCAF4/8 proteins bind the hyper-phosphorylated RNAPII C-terminal repeat domain (CTD) phosphorylated on both Ser2 and Ser5 and are detected at early, alternative polyA sites. Concomitant knockout of human *SCAF4* and *SCAF8* results in altered polyA selection and subsequent early termination, leading to expression of truncated mRNAs and proteins lacking functional domains and is cell lethal. While SCAF4 and SCAF8 work redundantly to suppress early mRNA termination, they also have independent, non-essential functions. SCAF8 is an RNAPII elongation factor, whereas SCAF4 is required for correct termination at canonical, distal transcription termination sites in the presence of SCAF8. Together, SCAF4 and SCAF8 coordinate the transition between elongation and termination, ensuring correct polyA site selection and RNAPII transcriptional termination in human cells.

## Highlights

• Human SCAF4 and SCAF8 couple RNAPII Ser2P- and Ser5P-binding and RNA processing
• SCAF4 and SCAF8 bind nascent RNA upstream of early polyadenylation sites
• SCAF4 and SCAF8 prevent early mRNA transcript cleavage and polyadenylation
• Lack of SCAF4 and SCAF8 result in truncated protein products and is cell lethal

## Graphical Abstract

**Figure:**
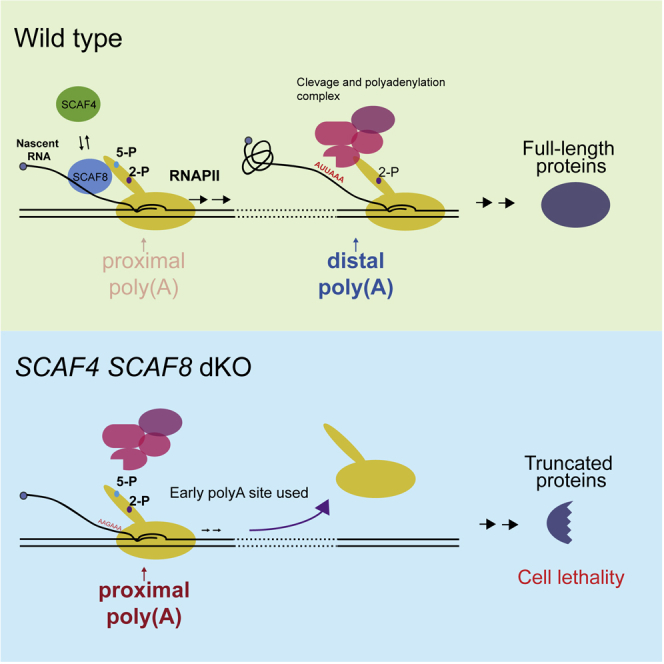

## Teaser

Eukaryotic anti-terminator proteins suppress usage of early polyadenylation sites to prevent production of truncated proteins.

## Introduction

RNA polymerase II (RNAPII) is responsible for transcription of all protein-coding genes and a number of non-coding RNAs. Whereas much work has focused on transcriptional initiation and its regulation, it is becoming increasingly clear that regulation of post-initiation events is crucial for gene expression as well. For example, co-transcriptional mRNA processing and the transition from elongating to terminating RNAPII, in particular, have emerged as significant points of regulation that still remain poorly understood (Proudfoot, 2016).

Looking to other branches of the evolutionary tree, it is clear that gene expression can be potently regulated via transcript termination. For example, the N protein of bacteriophage λ activates the lytic phase of phage development by suppressing the activity of transcriptional terminators that otherwise prevent phage protein synthesis in infected *Escherichia coli* cells. Anti-terminator proteins are encoded by the *E. coli* genome itself as well (Santangelo and Artsimovitch, 2011). Importantly, however, whereas the site of transcript termination in prokaryotes is determined by where RNAP disengages, the process consists of two coupled events in eukaryotes: cleavage and polyadenylation of the mRNA transcript, followed by RNAPII disassociation from the DNA template (i.e., transcriptional termination), which typically takes place a few kilobases downstream of the polyadenylation (polyA) site in mammalian cells. In eukaryotes, the 3′ end of the mRNA transcripts is thus dictated by the site of transcript cleavage, not by where RNAPII terminates transcription. Two, not necessarily mutually exclusive, models exist to describe RNAPII termination in eukaryotes. In the torpedo model, cleavage of the nascent transcript provides an entry point for the exonuclease XRN2 to degrade RNA attached to RNAPII from the 5′ end, which facilitates termination once it catches up with RNAPII (Connelly and Manley, 1988, Proudfoot, 2016). Alternatively, or additionally, the allosteric model posits that transcription through a functional polyA site brings about a conformational change in the RNAPII elongation complex, making it termination competent, which helps explains why transcript cleavage it not strictly required for termination *in vitro* (Edwalds-Gilbert et al., 1993, Kim and Martinson, 2003, Zhang et al., 2015). A common feature of both models is the recognition of polyA sites by the RNAPII complex as a prerequisite for termination.

Correct polyA site selection thus ensures correct maturation of the final mRNA transcript and plays a decisive role in determining the expression of a plethora of mRNA isoforms across the human genome. Intriguingly, the majority of human genes also express alternative, short mRNA isoforms, often of doubtful functional relevance (Zerbino et al., 2018). Indeed, it has been estimated that close to 70% of human genes utilize more than one polyA site, resulting in transcripts with varying coding or regulatory capacity or both (Derti et al., 2012). Because unwanted, early polyA site selection can have deleterious effects, aberrant transcripts originating from cryptic polyA sites must be suppressed through transcriptional quality-control mechanisms that remain poorly understood. Selection of cryptic, early polyA sites resulting in prematurely terminated mRNAs have been linked to disease (Elkon et al., 2013), and recently it was shown that widespread use of intronic polyA (IpA) sites in leukemia results in the expression of truncated proteins lacking the tumor-suppressive functions of the corresponding full-length proteins (Lee et al., 2018). Considering that higher eukaryotes often possess multiple polyA sites per gene, it would seem an obvious advantage to have evolved anti-termination factors to specifically regulate the usage of early polyA sites, but no candidate protein(s) for this critical role has so far been identified.

In eukaryotes, most mRNA-processing events are coupled to transcription through the C-terminal repeat domain (CTD) on the largest subunit of RNAPII, RPB1/POLR2A, which carries the consensus sequence Y^1^S^2^P^3^T^4^S^5^P^6^S^7^ (52 repeats in humans, and 26 in yeast) (Buratowski, 2009, Eick and Geyer, 2013). The phosphorylation pattern of the CTD changes dynamically during the transcription cycle to facilitate, or hinder, the recruitment of RNAPII co-factors, including numerous RNA-binding proteins that control the maturation of transcripts (Corden, 2013, Eick and Geyer, 2013, Pineda et al., 2015). Understanding the coupling between CTD phosphorylation and co-transcriptional mRNA processing remains a major challenge.

We sought to shed new light on co-transcriptional processes by focusing on the human SCAF4 and SCAF8 proteins. These proteins were initially discovered among a group of SR (serine-arginine rich), CTD-associated factors (SCAFs) uncovered in a yeast-two-hybrid screen for mammalian proteins that interact with the CTD of RNAPII (Yuryev et al., 1996). However, to date their molecular function remains largely unknown. The most closely related yeast orthologs of SCAF4 and SCAF8 are *Saccharomyces cerevisiae* Nrd1 and *Schizosaccharomyces pombe* Seb1. Whereas Nrd1 preferentially binds RNAPII via CTD Ser5P and regulates transcriptional termination of non-polyadenylated transcripts as part of the Nrd1-Nab3-Sen1 complex (Arigo et al., 2006, Vasiljeva et al., 2008, Schulz et al., 2013), Seb1 preferentially recognizes CTD Ser2P and promotes polyA site selection and termination at both protein-coding and non-coding genes (Lemay et al., 2016, Wittmann et al., 2017).

We used a multi-omic, genome-wide approach to investigate the function of SCAF4 and SCAF8 in human cells. Our data indicate that, while SCAF4 and SCAF8 have evolved interesting and important individual functions, their redundant, essential function appears to be as mRNA anti-terminators that suppress the use of early alternative polyA sites and thereby the accumulation of non-functional, truncated proteins.

## Results

**Figure 1:**
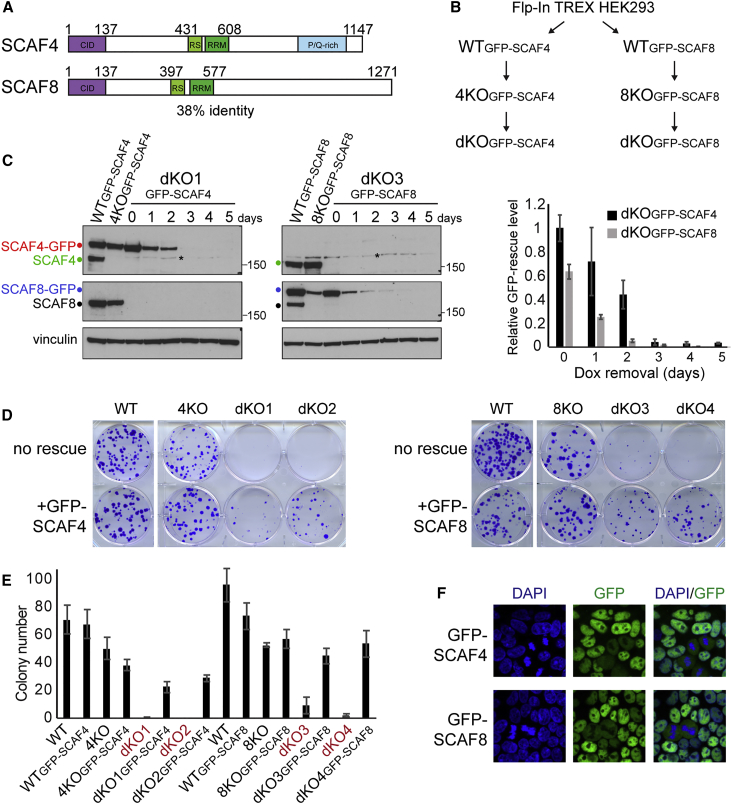
Double *SCAF4* and *SCAF8* Knockout Is Lethal (A) Domain structure of SCAF4 and SCAF8. (B) Scheme for generation of single and double *SCAF4* and *SCAF8* CRISPR knockouts (KOs). 4KO, single *SCAF4* KO; 8KO, single *SCAF8* KO; dKO, double KO. (C) Dox-inducible GFP-SCAF expression in WT and KO cell lines. Left, western blot before and after Dox removal. Asterisk between lanes for day 2 and 3 indicates non-specific band. Right, quantification of relative GFP-rescue protein levels. Error bars represent ±SD. (D) Colony formation assays, with cells grown either with (left, +GFP-SCAF4; right, +GFP-SCAF8) or without (no rescue) Dox for 5 days prior to seeding single cells for colony formation. (E) Quantification of colony formation assays from two biological replicates (each seeded in triplicate). Error bars represent ±SD. (F) Cellular localization of GFP-SCAF4 or GFP-SCAF8 in HEK293 cells. See also Figure S1.

**Figure S1:**
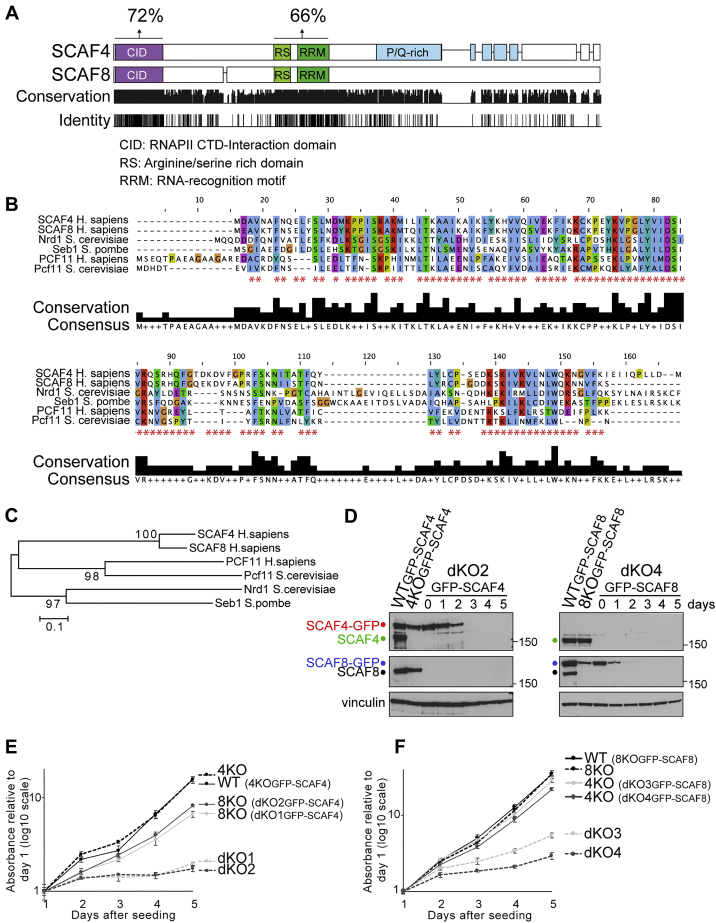
SCAF4 and SCAF8 Are Highly Similar, Functionally Redundant Proteins Containing a CID Also Found in Known Termination Factors, Related to Figure 1 (A) Alignment of SCAF4 and SCAF8 protein sequences showing identical and conserved residues below. Percentages above indicate identical residues between the SCAF4 and SCAF8 CID and RS/RRMs, respectively (B) Alignment of CIDs from SCAF4, SCAF8, Nrd1, Seb1, PCF11 and Pcf111. Red asterisks indicate residues that are identical between SCAF4 and SCAF8. (C) Phylogenetic tree (neighbor-joining) based on the CID alignment show in (B). (D) Verification of cell lines expressing a Dox-inducible GFP-SCAF4 or GFP-SCAF8 rescue in a WT-, single SCAF4 knockout- (*SCAF4* KO), single SCAF8 knockout- (*SCAF8* KO) or double knockout background (*SCAF4 SCAF8* dKO clone 2 [dKO2] or 4 [dKO4]). (E-F) Crystal violet-based cell proliferation assay in WT, *SCAF4* KO, *SCAF8* KO and dKO cell lines expressing either a GFP-SCAF4 rescue or GFP-SCAF8 rescue grown either with or without Dox for 5 days prior to seeding for growth assay. Error bars represent ± SD.

*SCAF4* and *SCAF8* likely arose via a gene duplication that occurred in vertebrates (Zerbino et al., 2018). The encoded proteins have significant sequence homology (38% identity and 50% similarity), and both contain a CTD-interaction domain (CID), characteristic of termination factors such as their orthologs in yeast, Nrd1 and Seb1, and termination factor PCF11 (Figures 1A and S1A–S1C). To study the function of SCAF4 and SCAF8, we used CRISPR technology to generate single *SCAF4* KO (4KO), single *SCAF8* KO (8KO), as well as double-knockout (dKO) cell lines, which also contained a single copy of a doxycycline (Dox)-inducible *SCAF4* or *SCAF8* rescue construct, with the encoded GFP-tagged protein expressed at near-endogenous levels (Figures 1B, 1C, and S1D). Cell lines were maintained in the presence of Dox to ensure expression of the CRISPR-resistant rescue gene during and after KO cell line generation. Removal of Dox resulted in the loss of the rescue protein: at days 3–5, SCAF4 or SCAF8 were undetectable (Figures 1C and S1D). At this time, the KOs did not exhibit any signs of stress, as indicated by normal cell-cycle profiles, and absence of DNA damage and apoptotic markers (data not shown). However, while the single KOs maintained normal proliferation rates, the *SCAF4 SCAF8* dKOs failed to proliferate (Figures S1E and S1F). Notably, dKO cells did not grow when seeded as single cells for colony formation (Figures 1D and 1E). Lethality could be rescued by the expression of either SCAF4 or SCAF8, suggesting that the encoded proteins share a common, essential function.

### SCAF4 and SCAF8 Interact with Ser2-Ser5 Bi-phosphorylated CTD

**Figure 2:**
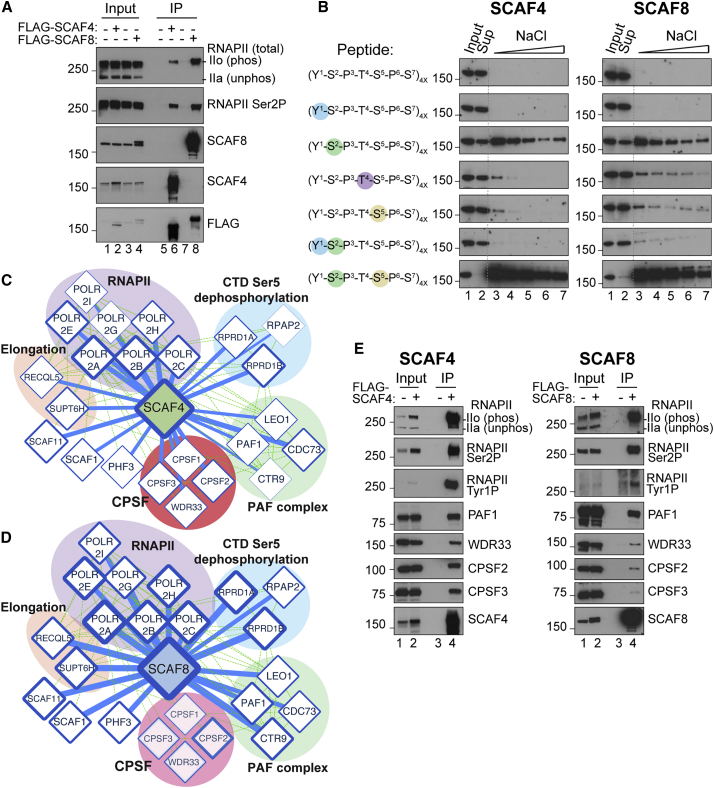
SCAF4 and SCAF8 Interact with Elongating RNAPII through Recognition of a Ser2-Ser5 Bi-phosphorylated CTD (A) IP of SCAFs from the chromatin fraction of cells expressing (or not) FLAG-tagged SCAF proteins. (B) SCAF binding assays using CTD peptides consisting of 4 heptad repeats, with phosphorylation at the indicated positions. NaCl washes (0.05, 0.2, 0.4, 0.8, and 1 M) were used to test the strength of binding. (C and D) Network analysis, using Cytoscape, of SCAF4 (C) and SCAF8 (D) interaction partners detected by label-free mass spectrometry of FLAG-IPs, using cells not expressing epitope-tagged protein as controls. The width of a connecting line represents average significance, while the width of the edge around the node represents average t test difference between the SCAF4 or SCAF8 IP and their control IP. (E) Western blot analysis of factors co-precipitating with FLAG-SCAF4 or FLAG-SCAF8. See also Figures S2 and S3 and Table S1.

**Figure S2:**
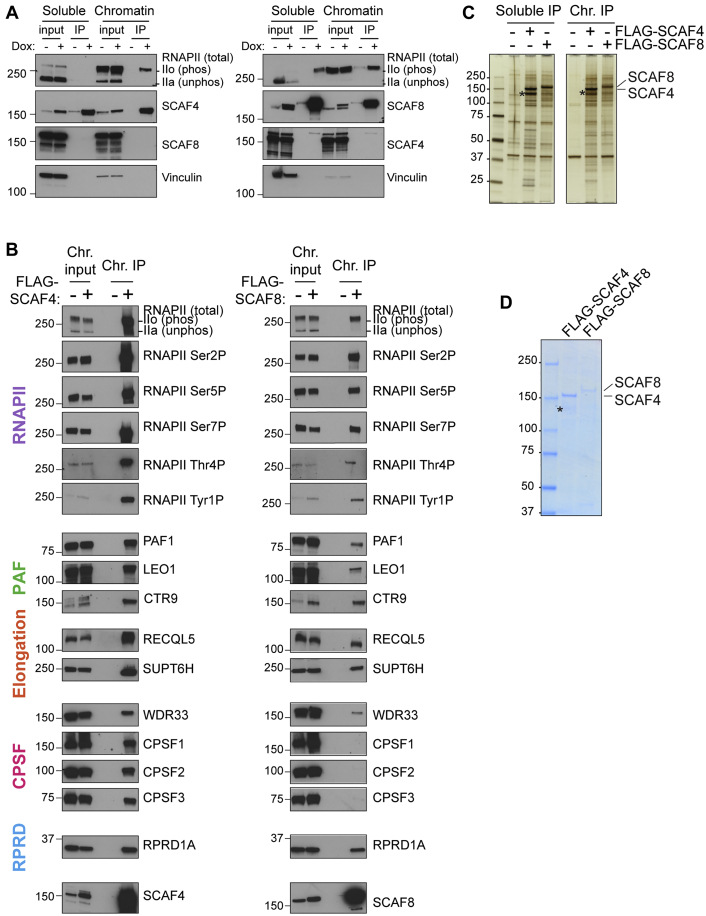
SCAF4 and SCAF8 Bind Phosphorylated RNAPII and RNAPII-Associated Proteins, Related to Figure 2 (A) FLAG-SCAF4 and SCAF8 IPs from soluble (cytoplasm + nucleoplasm) and chromatin enriched extracts from cells stably expressing Dox-inducible FLAG-tagged proteins. Non-induced FLAG-SCAF4 or FLAG-SCFA8 cell lines were used as negative controls. (B) SCAF4 and SCAF8 interaction with phosphorylated RNAPII and associated proteins. FLAG-SCAF4 or FLAG-SCAF8 IPs from chromatin enriched fractions from cells stably expressing FLAG-tagged SCAF4 or SCAF8. Cells not expressing a FLAG-tagged protein were used as control. (C) Silver stain of FLAG-purification of SCAF4 and SCAF8 from soluble (cytoplasmic and nucleoplasmic) and chromatin enriched fractions. FLAG IPs were washed with 150 mM NaCl containing buffer and FLAG peptide eluted. Asterisk indicate a degradation product of SCAF4 confirmed by western blotting. (D) Coomassie stain of FLAG-purification of SCAF4 and SCAF8 from chromatin enriched fractions. Extracts were pre-cleared prior to FLAG-IPs which were washed with 150 mM NaCl containing buffer and FLAG peptide eluted. Asterisk indicate a degradation product of SCAF4 confirmed by western blotting.

SCAF4 and SCAF8 are exclusively nuclear (Figure 1F), and immunoprecipitation (IP) experiments indicated that they both associate only with the transcriptionally engaged, hyper-phosphorylated form of RNAPII (Figures 2A and S2A). We thus detected Ser2P-, Ser5P-, Ser7P-, Thr4P-, and Tyr1P-modified RNAPII associated with SCAF4 and SCAF8 (Figure S2B). To investigate the potential direct recognition of phosphorylated CTD repeats, we examined binding of the SCAF proteins to chemically phosphorylated CTD peptides *in vitro* (Figure 2B). Purified, full-length SCAF proteins bound only to phosphorylated CTD peptides, with a very strong preference for peptides carrying Ser2-Ser5 double-phosphorylation and markedly less binding to peptides phosphorylated only at Ser2. Importantly, double-phosphorylation at Tyr1 and Ser2 markedly reduced binding relative to Ser2 phosphorylation alone (Figure 2B), suggesting that the strong preference for Ser2-Ser5 bi-phosphorylation is position specific and not simply due to the increased negative charge of double-phosphorylated repeats. Ser5P RNAPII is generally enriched in the beginning of genes, whereas Ser2P RNAPII levels increase through the gene and peak around the termination site (Hintermair et al., 2012, Voss et al., 2015). Given our expectation that SCAF4 and SCAF8 would be termination factors, these results were at first glance surprising; they were, however, in line with results from binding experiments using a SCAF8 CID fragment (Patturajan et al., 1998, Becker et al., 2008).

### SCAF4 and SCAF8 Are Associated with Elongating and Terminating RNAPII Complexes

**Figure S3:**
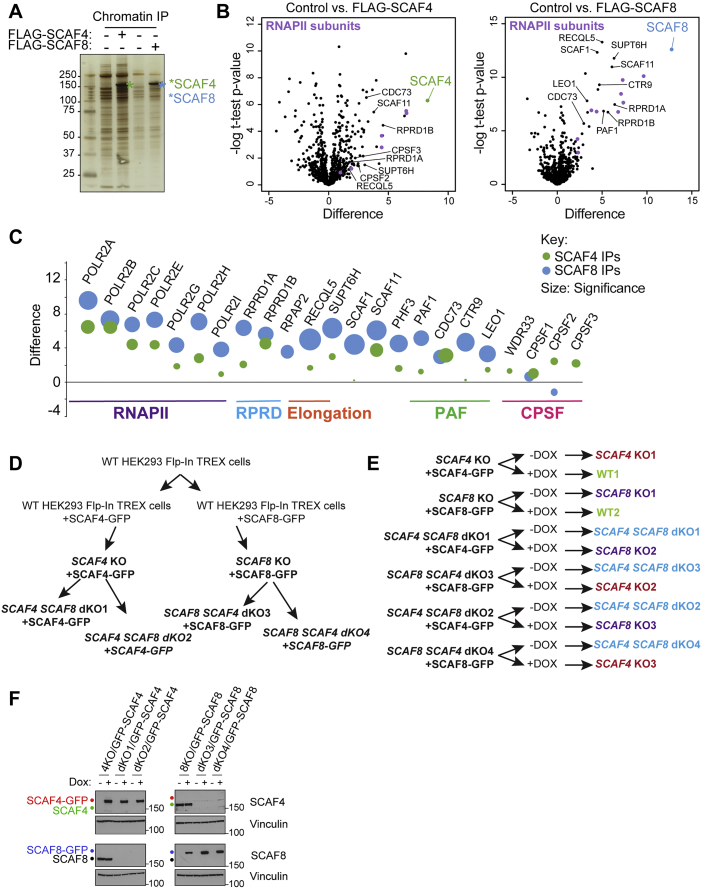
SCAF4 and SCAF8 Interactomes and Cell Lines Used for RNA Sequencing Experiments, Related to Figures 2 and 3 (A) Silver stains of FLAG-SCAF4 and FLAG-SCAF8 IPs from chromatin enriched fractions from cells stably expressing FLAG-tagged SCAF4 or SCAF8 used for mass-spectrometry. Cells not expressing FLAG-tagged proteins were used as controls. (B) Volcano plots of FLAG-SCAF4 (left) and FLAG-SCAF8 (right) IP mass-spectrometry results. RNAPII subunits are indicated in purple. Data represent t test significance scores and differences considering data from 2 biological replicates each measured from triplicate injections. (C) Bubble plot of t test differences (2 biological replicates each injected as three technical injections) between FLAG-SCAF4 (green) or FLAG-SCAF8 (blue) and control IPs for indicated proteins. Bubble size is proportional with significance score for the indicated proteins. (D) Schematic outline of CRISPR knockout cell lines generated in this study, with cell lines used for RNA sequencing experiments highlighted in bold. (E) Overview of 6 different cell lines used for RNA sequencing experiment. Each cell line was grown for 5 days with or without Dox resulting in the genotypes shown on the right-hand side. (F) Western blot validation of cell lines used for TT-Seq (replicate 1). Whole cell extracts harvested at day 5 after Dox wash out in parallel with RNA for sequencing experiments. Similar validation was carried out for every RNA-Seq experiment to confirm efficient removal of the GFP-tagged rescue proteins *SCAF4* KO is abbreviated 4KO and *SCAF8* KO is abbreviated 8KO.

Although SCAF4 and SCAF8 recognize the same form of the RNAPII CTD, we never detected SCAF4 in SCAF8 IPs, or vice versa, indicating that they cannot bind the same RNAPII complex (Figures 2A and S2A), and opening the possibility that they might both functionally compete *and* complement each other. Purification indicated that SCAF4 and SCAF8 are not part of stable complexes with other subunits (Figures S2C and S2D). To identify interaction partners, we used label-free quantitative mass spectrometry to analyze FLAG-SCAF IPs derived from solubilized chromatin (Figure S3A). The strongest interactor for both SCAF4 and SCAF8 was RNAPII (Figures S3B and S3C). In addition, we identified subunits from the PAF complex, the RPRD1-RPAP2 complex involved in Ser5P dephosphorylation (Ni et al., 2014), the elongation factors SUPT6H (also known as SPT6) and RECQL5, as well as largely uncharacterized, RNAPII-associated proteins such as PHF3 (Figures 2C, 2D, S2B, S3B, and S3C; Table S1). We note that while SCAF4 and SCAF8 failed to interact with each other, the CTD-associated SCAF1 and SCAF11 proteins were consistently found as interactors of both factors, presumably through a common interaction with RNAPII, opening the possibility that more than one SCAF protein (but not SCAF4 and SCAF8) can bind RNAPII at the same time, and suggesting that SCAF4 and SCAF8 might compete for the same binding surface on the body of RNAPII (see Discussion for details). Interestingly, we failed to detect SETX (human ortholog of *S. cerevisiae* Sen1) and components of the RNA exosome (Table S1), which interact with the SCAF4/8 ortholog Nrd1 in budding yeast (Vasiljeva and Buratowski, 2006). Taken together, these interactomes support the idea that SCAF4 and SCAF8 are associated with RNAPII elongation complexes.

Interestingly, the SCAF4/SCAF8 interactors also included 3′ end-processing factors, detected most prominently in SCAF4 IPs, suggesting that the SCAFs might also associate with terminating RNAPII (Figures 2C–2E and S3C). These processing factors included members of the cleavage and polyadenylation specificity factor (CPSF) complex, CPSF1, CPSF2, CPSF3, and WDR33. Western blot analysis confirmed these interactions (Figures 2E and S2B).

### In Contrast to Nrd1, SCAF4 and SCAF8 Do Not Restrict Anti-sense, Pervasive, Non-coding Transcription

**Figure 3:**
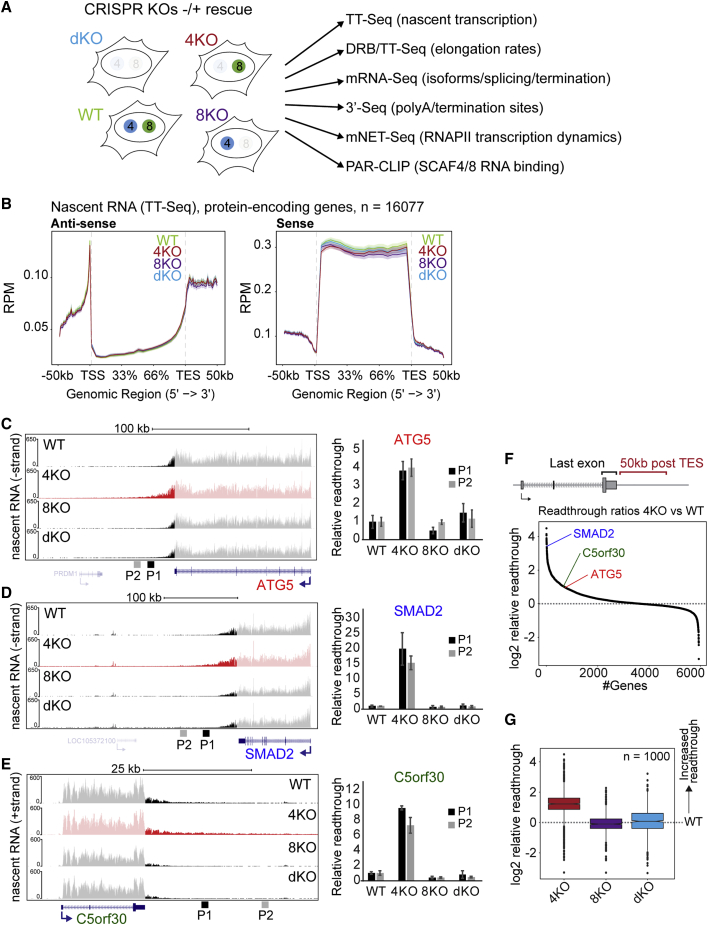
SCAF4 Suppresses Transcriptional Readthrough of Protein-Encoding Genes (A) Cell lines and genome-wide techniques used to assess SCAF4 and SCAF8 function. (B) Metagene profiles for strand-specific TT-seq (nascent RNA sequencing [RNA-seq]). (C–E) Left, UCSC genome browser view of TT-seq for (C) *ATG*5, (D) *SMAD2*, and (E) *C5orf30*. Right, qPCR quantification of transcriptional readthrough (primer pairs P1 and P2, see left side), relative to *GAPDH* and an internal intron-spanning area, normalized to WT. Error bars represent ±SD (F) Readthrough ratios calculated as TT-seq reads in a region 50 kb downstream of the TES relative to reads in the terminal exon, in *SCAF4* KO versus WT cells, ranked according to ratio. (G) Boxplot of readthrough ratios for the 1,000 most affected genes calculated relative to WT. See also Figures S3 and S4 and Table S2.

In order to examine the effect of SCAF4 and SCAF8 on gene expression, we used the KO cell lines and a wide variety of genome-wide techniques (Figure 3A). Due to the manner in which these cell lines were generated (see Figures 1B, S3D, and S3E), a total of 12 cell types (6 different cell lines grown with or without Dox) were often analyzed together. For example, dKOs were generated either by first knocking out *SCAF4* and then *SCAF8*, or vice versa, and these different cell lines were in turn derived from cell lines containing either a Dox-inducible *SCAF4* or a *SCAF8* rescue gene, giving rise to a total of 4 genotypically identical *SCAF4 SCAF8* dKO cell lines (Figures S3D and S3E). Moreover, a dKO that expresses a rescue gene (i.e., grown in the presence of Dox) is effectively a single KO; for example, a dKO expressing the *SCAF4* rescue gene is genotypically and phenotypically a *SCAF8* single KO cell line (See Figures 1D and S3D–S3F).

**Figure S4:**
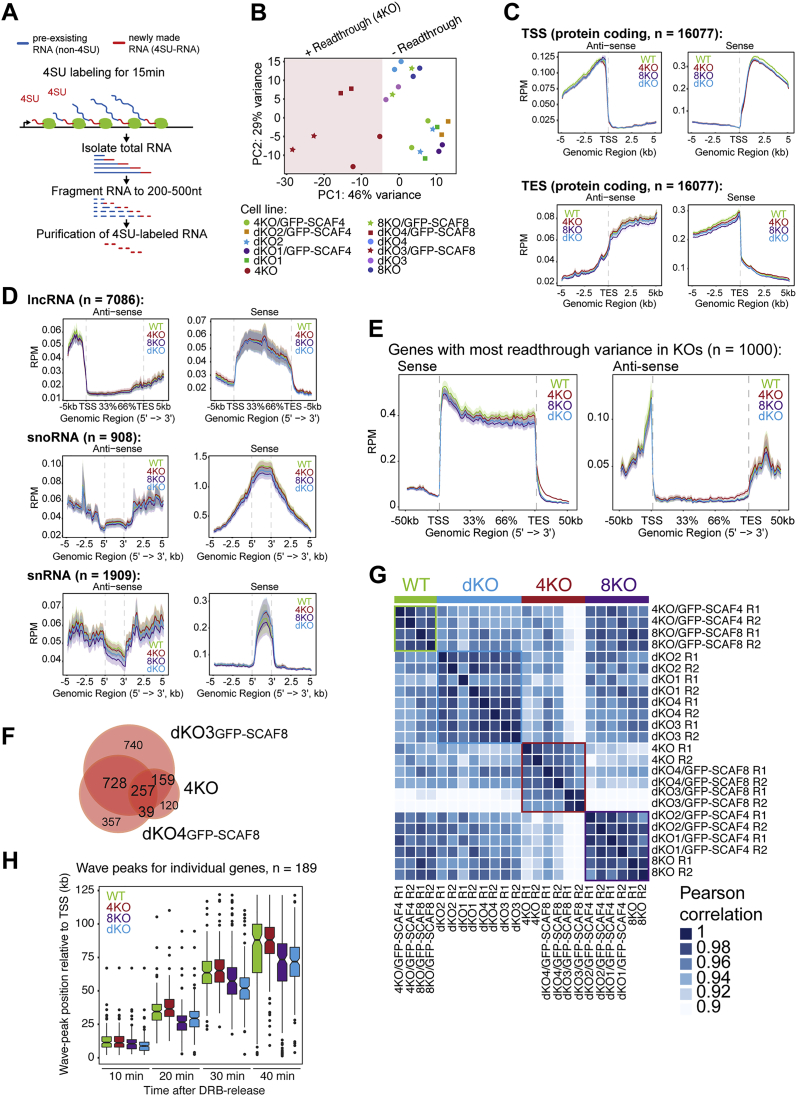
TT-Seq and DRB/TT-Seq in *SCAF4* and/or *SCAF8* KOs, Related to Figures 3 and 4 (A) Outline of TT-Seq to measure nascent RNA transcription. Cells were labeled with 1 mM 4SU for 15 min. Total RNA isolated, fragmented to 200-500 nt, 4SU residues biotinylated and purified using streptavidin beads to separate pre-existing non-labeled RNA from biotinylated 4SU-labeled newly synthesized RNA. 4SU-RNA is used for strand-specific library preparation and high-throughput sequencing. (B) Principal component analysis (PCA) of the 24 individual TT-Seq samples. *SCAF4 KO* cells with transcriptional readthrough are highlighted. Each cell line was sequenced from two biological replicate experiments, resulting in 4X WT samples (2 cell lines x 2 biological replicates), 6X *SCAF4* KO samples (3 cell lines x 2 biological replicates, 6X *SCAF8* KO samples (3 cell lines x 2 biological replicates) and 8X dKO samples (4 cell lines x2 biological replicates). (C) TT-Seq profiles around the TSS and TES of protein coding genes. (D) Metagene profiles for lncRNA, snoRNA and snRNA including the 5 kb upstream and downstream regions. (E) Metagene profile for the 1000 genes with the highest variation of readthrough ratios between any CRISPR group (*SCAF4* KO (4KO), *SCAF8* KO (8KO) or dKO and WT. (F) Overlap of readthrough genes between the three *SCAF4* KO cells lines (*SCAF4* KO, dKO3/GFP-SCAF8 and dKO4/GFP-SCAF8) with a relative readthrough ratios above 1.5 in two biological replicates of the indicated cell line (*SCAF4* KO versus *SCAF4* KO/GFP-SCAF4, dKO3 versus dKO3 versus dKO3/GFP-SCAF8 and dKO4 versus dKO4/GFP-SCAF8). (G) Correlation plot of readthrough ratios for the top 1000 genes with the largest coefficients of variation in their readthrough ratios across all individual 24 samples. WT (green), *SCAF4* KO (red), *SCAF8* KO (purple) and dKO (blue) cell lines are indicated. (H) RNAPII elongation wave peak positions for individual genes (> 60 kb) calculated from DRB/TT-Seq data following DRB release. Please note that the variation of the 40 min wave peak positions in some cases are caused by RNAPII termination downstream of the TES.

To monitor nascent transcription, we used a short pulse of 4-thiouridine (4SU) to label newly synthesized transcripts (Figure S4A). To obtain high-resolution profiles, an RNA fragmentation step was employed, similar to in transient transcriptome sequencing (TT-seq) (Schwalb et al., 2016) (Figure S4A). Although our protocol uses a different fragmentation method, we will refer to it as TT-seq for simplicity. This approach gave rise to high-quality data sets for which principal component analysis showed that most of the variation between samples from the 12 different conditions was indeed explained by “genotypic” status (Figure S4B). For simplicity, we thus merged experiments based on their genotype (e.g., data for *SCAF4* KO were derived from *SCAF4* KO, dKO1/GFP-SCAF8, and dKO2/GFP-SCAF8 together). Metagene analysis of sense and anti-sense transcription for protein-encoding genes showed remarkably similar profiles overall (Figure 3B). Importantly, we did not observe an increase in divergent, anti-sense transcription originating near the transcription start site (TSS) of protein-encoding genes (Figures 3B and S4C), and we also failed to detect differences in either sense or anti-sense transcription for long non-coding RNAs (lncRNAs), small nuclear RNAs (snRNAs), or small nucleolar RNAs (snoRNAs) (Figure S4D). These results suggest that, in contrast to Nrd1 in *S. cerevisiae*, SCAF4 and SCAF8 do not serve to restrict anti-sense transcription or terminate snRNA or snoRNA genes in human cells.

### SCAF8 Is a Positive RNAPII Elongation Factor and Promotes Transcriptional Readthrough in the Absence of SCAF4

Interestingly, for a subset of genes, an increase in nascent transcripts up to 50–100 kb downstream of the transcription end site (TES) was detected, specifically in the *SCAF4* KO (Figures 3C–3G, S4E, and S4F). The termination window downstream of the TES has a median length of ∼3,500 bp in human cells (Schwalb et al., 2016), suggesting that RNAPII in *SCAF4* KO cells continue transcription beyond normal termination sites, also known as transcriptional readthrough (Rutkowski et al., 2015, Vilborg et al., 2015). Indeed, most of the variance in the TT-seq data was observed between this KO (which had increased RNA levels downstream of canonical termination windows) and the remaining cell types (which did not) (Figures S4B and S4G). Many genes (n = 1,281) displayed ≥1.5-fold increase in nascent RNA in a 50 kb region downstream of the TES (Table S2). Importantly, we did not detect elevated transcript levels in the *SCAF4 SCAF8* dKOs (Figures 3C–3E and 3G), indicating that terminator readthrough is unlikely to cause lethality in the dKOs and also showing that such readthrough depends on SCAF8. Although other explanations cannot be ruled out, these results agree with a model in which SCAF4 and SCAF8 compete for the same RNAPII complex: in the absence of SCAF4, SCAF8 may thus freely associate with RNAPII at the TES to bring about transcriptional readthrough.

**Figure 4:**
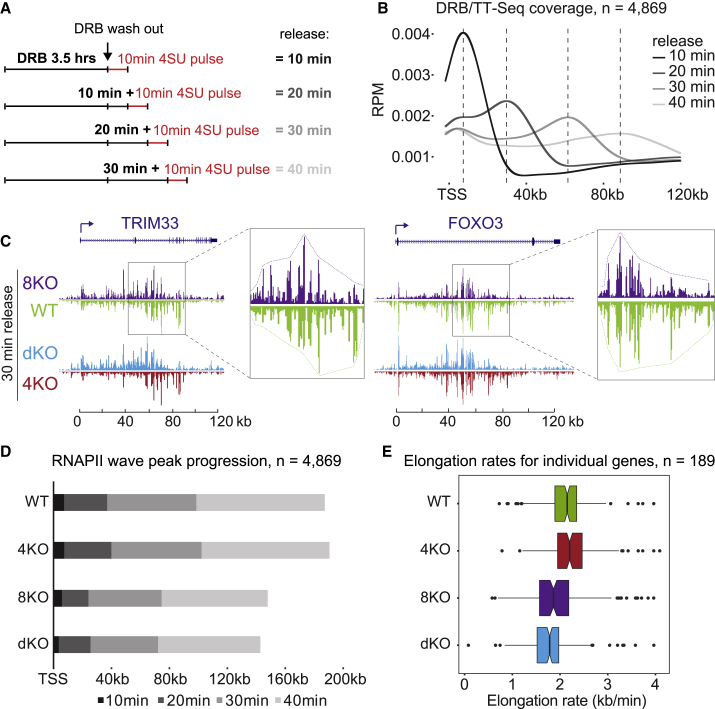
SCAF8 Promotes Transcript Elongation (A) Schematic of DRB/TT-seq to measure RNAPII elongation rates. (B) WT metagene profiles of DRB/TT-seq for genes 60–300 kb long. Vertical dashed lines indicate wave peak positions. (C) UCSC genome browser views of DRB/TT-seq results 30 min after DRB release at *TRIM33* and *FOXO3*. Notice that the differences highlighted by stippled boxes and enlargements are also seen in dKO (light blue), but not *SCAF4* KO (red). *TRIM33 and FOXO33* are not subject to changes in polyA site selection. (D) Cumulative wave peak progression from metagene analysis. (E) RNAPII elongation rates for individual genes with high DRB/TT-seq coverage across all cell lines and time points. Elongation rates based on metagene, wave-front analysis (n = 4,869 genes) were similarly: 2.3 (WT), 2.3 (*SCAF4* KO), 1.9 (*SCAF8* KO), and 1.8 kb/min (dKO). See also Figure S4.

Previous results suggested kinetic competition between elongation and termination, such that the rate of elongation affects termination (McDowell et al., 1994, Fong et al., 2015). To determine whether SCAF4 or SCAF8 influences the RNAPII elongation rate, we used the CDK9 and transcription elongation inhibitor DRB in combination with TT-seq, hereafter called DRB/TT-seq, as a variant of DRB/global run-on sequencing (GRO-seq) (Saponaro et al., 2014). In DRB/TT-seq, the position of RNAPII in the body of genes is analyzed by TT-seq at different times after removing DRB to release RNAPII from promoter-proximal gene areas (Figures 4A and 4B). These experiments revealed that SCAF8, but not SCAF4, positively affects the RNAPII elongation rate. Indeed, in both *SCAF8* KO and *SCAF4 SCAF8* dKO, elongation rates were reduced compared to WT and *SCAF4* KO cells, as evident from both single gene profiles and metagene analysis (Figures 4C–4E and S4H). The average elongation rates calculated for a group of long genes were 2.2 kb/min in both WT and *SCAF4* KO, compared to 1.9 kb/min in *SCAF8* KO and 1.8 kb/min in dKO (Figure 4E). Given that slow elongation was observed in both *SCAF8 KO* and the dKOs, and *SCAF8 KO* cells are viable, it seems implausible that slow RNAPII elongation is the cause of lethality in the dKO cell lines.

Together, the data above support the idea that SCAF4 and SCAF8 have a redundant, essential function, but also that they have distinct roles in transcription. SCAF8 thus acts a positive elongation factor to promote efficient progression of RNAPII through genes, whereas SCAF4 restricts transcriptional readthrough at a large number of genes. We suggest that such transcriptional readthrough fails to occur in the *SCAF4 SCAF8* dKO because RNAPII transcript elongation proceeds at a slower rate in the absence of SCAF8, which in this cell line allows termination even in the absence of SCAF4.

### A Common Function for SCAF4 and SCAF8 as Transcriptional Anti-terminators

**Figure 5:**
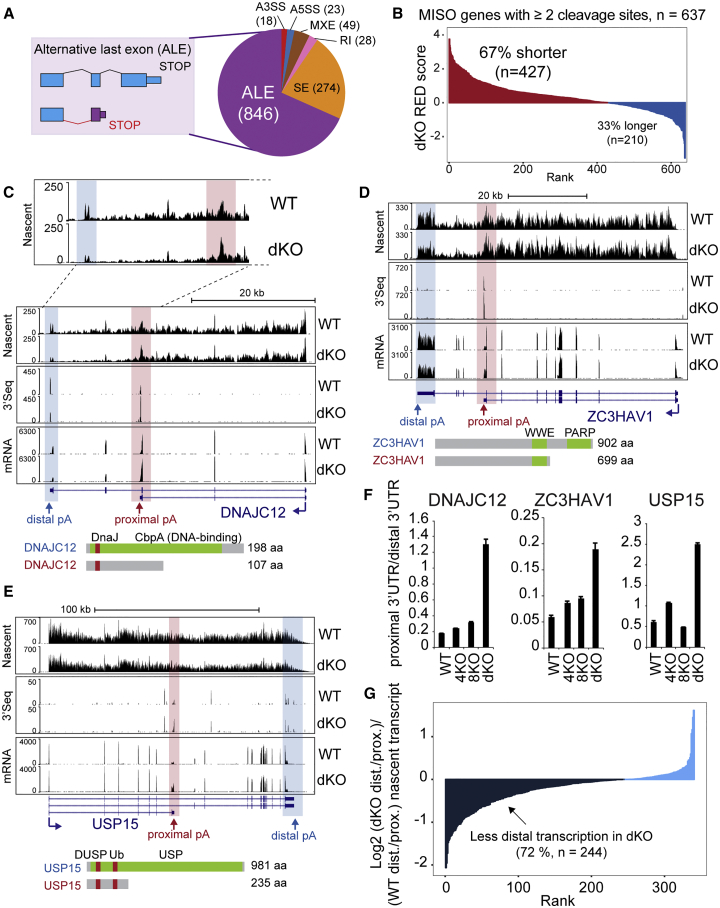
*SCAF4 SCAF8* dKO Affects Alternative Polyadenylation and Termination (A) mRNA isoform expression changes in the dKO detected by MISO analysis of mRNA-seq (Bayes factor ≥10 and dPSI ≥ ±0.3). A3SS, alternative 3′ splice site; A5SS, alternative 5′ splice site; MXE, mutually exclusive exons; RI, retained intron; SE, skipped exon; ALE, alternative last exon. (B) Relative expression differences (RED) in dKO for genes with mRNA isoform changes by MISO analysis and containing ≥2 high-confidence cleavage sites by 3′-seq. (C–E) UCSC genome browser tracks comparing nascent RNA (TT-seq), mature mRNA (mRNA-seq), and polyA sites (3′-seq) in dKO and WT cells for DNAJC12 (C), ZC3HAV1 (D), and USP15 (E). Protein products with annotated domains are indicated below. The short isoforms lack predicted functional domains. (F) qPCR validation of RNA-seq data. Graphs show ratios of proximal to distal 3′ UTR, normalized to *GAPDH* and a gene-specific, intron-spanning primer-pair reference common to the two isoforms. Error bars represent ±SD. (G) Nascent transcription at the most distal versus most proximal terminal exon (TT-seq signal) for SCAF4- and SCAF8-regulated genes (significant MISO ALE event in WT versus dKO) with at least 2 high-confidence annotated transcripts with TT-seq signal for both terminal exons (n = 340). See also Figures S5 and S6 and Table S2.

**Figure S5:**
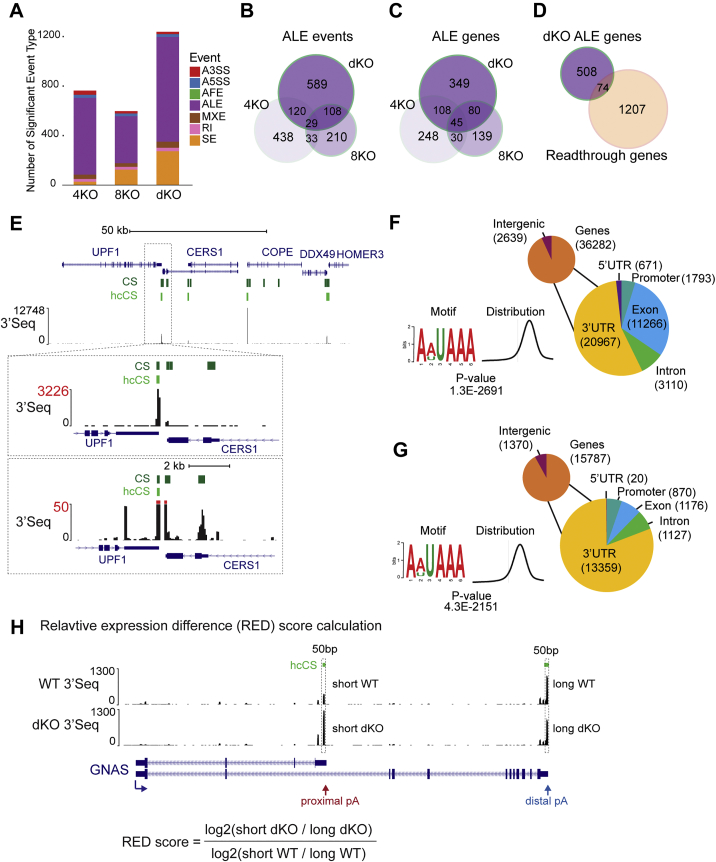
Identification of polyA Site Changes in *SCAF4* and *SCAF8* KOs, Related to Figure 5 (A) Significant MISO events (bayes factor ≥ 10 & dPSI ≥ +/−0.3) identified in *SCAF4* KO, *SCAF8* KO and *SCAF4 SCAF8* dKO compared to WT. Event types are abbreviated as: A3SS: Alternative 3′ splice sites, A5SS: Alternative 5′ splice sites, MXE: Mutually exclusive exons, RI: Retained introns, SE: Skipped exons, ALE: Alternative last exons. (B) Overlap of significant ALE events between *SCAF4* KO, *SCAF8* KO and dKO (C) Overlap of genes harboring significant ALE event in *SCAF4* KO, *SCAF8* KO and dKO. (D) Overlap of genes with an ALE event in dKO cells and genes with transcriptional readthrough in *SCAF4* KOs. (E) UCSC genome browser view of identified cleavage sites (CS) and high-confidence cleavage sites (hcCS) in the *UPF1-HOMER3* locus. (F-G) Position of identified of cleavage sites (F) and high-confidence cleavage sites (G) from 3′-Seq mapping to intergenic regions or within annotated Ensembl genes. The most significantly enriched motif in a 50 bp up- and downstream regions of both sets of cleavage-sites and the location relative to the cleavage site using MEME are shown to the left. (H) Outline of relative expression (RED) score calculation used to quantify changes in mRNA transcript cleavage based on 3′Seq data.

Co-transcriptional events such as splicing, polyA site selection, and termination are highly regulated and dictate mRNA isoform expression in a number of ways (Bentley, 2014). To determine whether and how *SCAF4* and *SCAF8* knockout influences mRNA splicing and polyA site selection, we used the mixture-of-isoforms (MISO) model (Katz et al., 2010) on mRNA sequencing (mRNA-seq) data from the different KO cell lines. MISO analysis is typically used to highlight changes to mRNA splicing, such as altered inclusion, or exclusion, of specific exons. Somewhat surprisingly, none of the *SCAF* KO cells lines showed marked changes to mRNA splicing compared to wild-type. Instead, the most dramatic change was observed in *SCAF4 SCAF8* dKO cells, which showed altered use of alternative last exons (ALEs), representing almost 70% of all events (846 out of 1,238 total) (Figure 5A; Table S2). Crucially, ALEs are mechanistically the result of alternative polyA site usage, creating a new terminal exon and in turn dictating mRNA termination (Figure 5A, left) (Elkon et al., 2013). Alternative polyA signals are often located within intronic regions of longer transcript isoforms and have therefore also been named IpA sites (Singh et al., 2018). Besides the ALE events observed in the dKO, we also observed a smaller number of ALE events in the single *SCAF4* or *SCAF8* KOs, but, interestingly, most of these did not overlap with the ALE events detected in the dKO (Figures S5A–S5C). More importantly, of the 1,281 genes previously found to display signs of transcriptional readthrough of canonical, distal termination sites in *SCAF4* KO cells (see Figure 3 and Table S2), only 74 also had an ALE event in dKO cells (Figure S5D), showing that the genes with alternative polyA site selection in the dKO were *not* identical to those that had transcriptional readthrough at distal, canonical terminators in *SCAF4* KO cells. This further supports the idea that, depending on the context, SCAF4 and SCAF8 may either compete or functionally complement each other.

**Figure S6:**
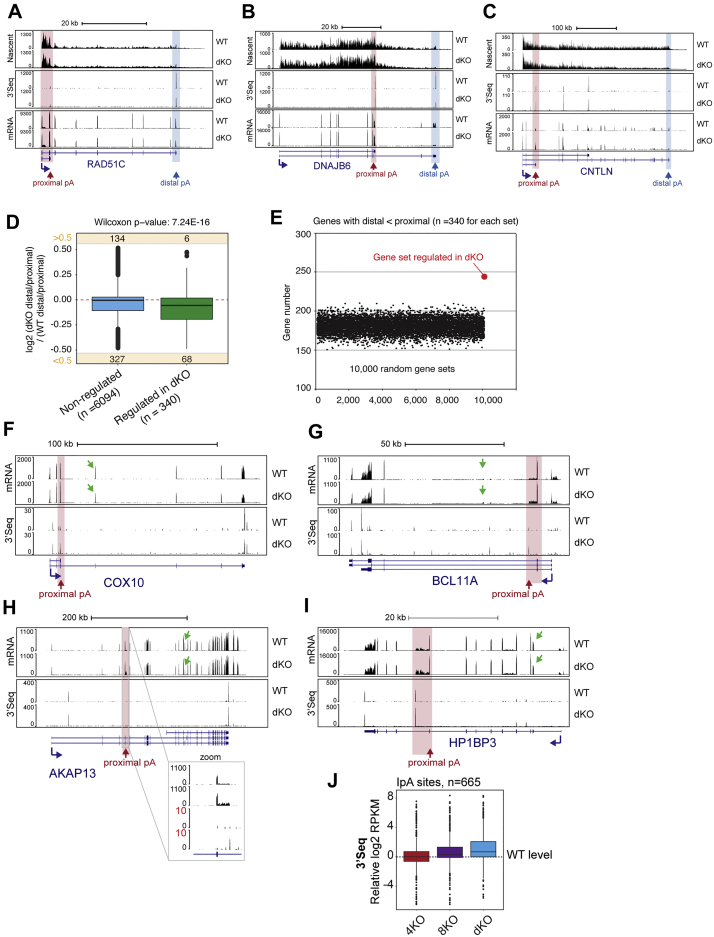
Early polyA Site Selection in *SCAF4 SCAF8* dKOs Is Accompanied by Early Transcriptional Termination, Related to Figure 5 Genome browser tracks showing nascent RNA (TT-Seq), mRNA-Seq and 3′-Seq for (A) *RAD51C*, (B) *DNAJB6* and (C) *CNTLN* in dKO and WT cells. (D) Boxplot of nascent transcription (TT-Seq) for distal and proximal terminal exons in SCAF regulated genes (significant ALE event in WT versus dKOs, n = 340) and non-regulated genes with > 2 terminal exons (n = 6094). (E) Number of genes with less nascent (TT-Seq) transcription in their distal terminal exon compared to the proximal terminal exon for 10,000 randomly selected genes set (all n = 340) compared to the SCAF4 and SCAF8 regulated gene set (n = 340). (F-I) Genome browser tracks showing examples of genes with skipped exon (SE) events identified by MISO (affected exons as detected by MISO is indicated by green arrows) in *COX10* (F), *BCL11A* (G), *AKAP13* (H) and *HP1BP3* (I). (J) Relative usage of intronic polyadenylation (IpA) sites identified by Singh et al.*,* 2018 in *SCAF4* KO (4KO), *SCAF8* KO (8KO) and dKO (dKO). Only IpA sites with a total RPKM ≥ 50 across all samples were considered.

To directly measure changes to 3′ end processing in the *SCAF* KO cells, we performed 3′ sequencing (3′-seq), which quantitatively captures the usage of polyA sites genome-wide (Elkon et al., 2013). Confirming the quality of the resulting data, the majority of detected cleavage sites were within the 3′ UTRs of annotated genes (see example in Figure S5E), with the most significantly recurring motif being the canonical polyA site (AAUAAA) located 10–35 nt upstream of the transcript cleavage site (Figures S5F and S5G). To determine whether the genes displaying mRNA isoform changes by MISO analysis *also* displayed alternative polyadenylation changes in our 3′-seq data, we calculated relative expressing difference (RED) (Li et al., 2015) for genes containing 2 or more high-confidence cleavage and polyA sites as detected by 3′-seq. The RED score thus captures differential usage of polyA sites within individual transcripts (Figure S5H; Table S2; see also STAR Methods for details). Strikingly, using this approach a clear shift toward the use of more proximal polyA sites was observed in *SCAF4 SCAF8* dKO cells (Figure 5B), in support and extension of the data from MISO analysis. Gene examples with a preference for a more proximal polyA site usage in the dKO included *DNAJC12*, *USP15*, and *ZC3HAV1* (Figures 5C–5F; further examples in Figures S6A–S6C). Because 3′-seq generally detected polyadenylated 3′ ends corresponding to the shorter mRNA isoforms detected by mRNA-seq, the shorter mRNA isoforms are caused by the changes in polyA site selection.

We now investigated whether the change in polyA site selection might in turn result in premature transcriptional termination downstream of the early polyA sites. Analysis of the TT-seq data supported this contention. Among the individual examples in Figure 5, this was most clearly visible in the *DNAJC12* gene (Figure 5C, top, cf. read peaks in red and blue squares), but it was observed in the other examples as well (Figures 5D and 5E) and more generally across the group of ALE genes regulated in a SCAF4/SCAF8-dependent manner (Figures 5G, S6D, and S6E). The effect was specific to genes in which polyA site selection was altered in the dKOs and not a general effect on genes with multiple isoforms (Figures S6D and S6E). Together, these data indicate that the marked shift toward utilizing early polyA sites in *SCAF4 SCAF8* dKOs is generally accompanied by subsequent termination of RNAPII transcription downstream.

One limitation with computational analysis of mRNA isoform changes is that it relies entirely on annotated events, with a recent study suggesting that IpA sites are much more widespread than previously appreciated (Singh et al., 2018). Thus, the 846 examples of early alternative polyA site selection we detected are likely to be an underestimate of the actual number of such events in the dKOs. Indeed, visual inspection of other mRNA isoform changes taking place in the dKOs indicates that many of the events that were called by MISO as “non-ALE events” were actually caused by changes in polyA sites as well, but that these were not classified as ALEs due to incorrect or lacking annotation (Figures S6F–S6I). To further address this issue, we took advantage of a collection of IpA sites recently identified via 3′-seq in different human cells and tissues (Singh et al., 2018). Tellingly, our 3′-seq data showed that *SCAF4 SCAF8* dKOs have increased usage of no less than 69% of that in Singh et al. (2018). IpA sites that were also detectable in our cell lines (Figure S6J; Table S2). Notably, 74% of these sites (339 out of the 456 sites with increased IpA usage) were accompanied by increased short mRNA isoform expression as well (Table S2).

Taken together, the data above indicate that concomitant deletion of *SCAF4* and *SCAF8* results in a dramatic increase in the use of proximal, alternative polyA sites in at least 1,300 genes across the human genome (see also below), often resulting in premature transcriptional termination downstream. The data also support the idea that SCAF4 and SCAF8 complement each other’s function at alternative polyA sites: at least one of the two factors is required for suppressing early polyA site selection.

### SCAF4 and SCAF8 Bind Nascent RNAPII Transcripts

The data above indicate an important, redundant role for SCAF4 and SCAF8 as mRNA anti-terminators, but whether this role is direct remained unclear. We therefore next sought to characterize the localization of SCAF4 and SCAF8 across the genome, to establish whether they are present at the transcripts and genes they affect. To study the RNA-binding pattern of SCAF4 and SCAF8, we mapped their binding sites, transcriptome-wide, using a modified photoactivatable ribonucleoside-enhanced crosslinking and immunoprecipitation (PAR-CLIP) protocol (see STAR Methods) (Hafner et al., 2010). This method involves incorporation of 4SU into nascent RNA followed by irradiation at 365 nm wavelength to induce crosslinking of RNA-binding proteins to 4SU-labeled RNA. IP of the respective SCAF proteins was then followed by isolation of the associated RNA, which was digested prior to preparation of libraries (enriched for insert sizes between 20 and 80 nucleotides) and deep sequencing. Sites of 4SU-protein crosslinking were detected as clusters of T→C transitions that arise because crosslinking causes an ∼5-fold increase in the frequency of G mis-incorporation opposite crosslinked 4SU during cDNA synthesis (Hafner et al., 2010).

**Figure S7:**
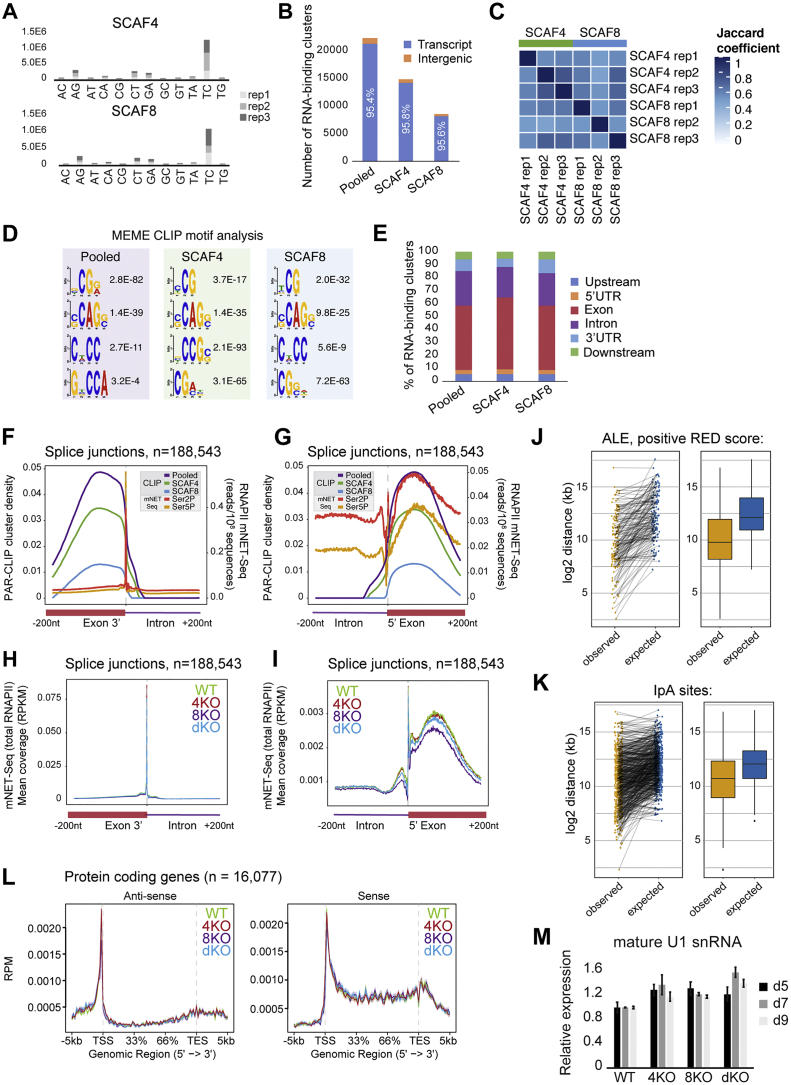
RNA-Binding Pattern of SCAF4 and SCAF8, Related to Figures 6 and 7 (A) Number of uniquely mapping PAR-CLIP reads containing nucleotide edits in SCAF4 (top) or SCAF8 (bottom) pull-outs. (B) Number of RNA-binding clusters mapping to annotated genes (including 2 kb upstream of TSS and 2 kb downstream of TES) or intergenic regions. Pooled clusters are defined as clusters overlapping between ≥ 2 out of 6 SCAF4 or SCAF8 PAR-CLIP experiments. SCAF4 clusters are clusters found in ≥ 2 out of 3 SCAF4 PAR-CLIP experiments. SCAF8 clusters are clusters found in ≥ 2 out of 3 SCAF8 PAR-CLIP experiments. (C) Jaccard correlation of CLIP cluster overlap between individual SCAF4 and SCAF8 replicates. (D) MEME motif analysis of PAR-CLIP binding sites. The four most significant motifs for the pooled, SCAF4 and SCAF8 CLIP cluster sets, sorted according to similarity. (E) Genomic localization of clusters mapping to annotated genes calculated from the cluster midpoint. (F-G) SCAF4 and SCAF8 cluster distribution in the ± 200 nt region around exon-intron (F) or intron-exon (G) junctions (n = 188,543). RNAPII Ser2P and Ser5P mNET-Seq profiles from Nojima et al. are shown as a reference. (H-I) mNET-Seq (total RNAPII) coverage in the ± 200 nt region around exon-intron (H) or intron-exon (I) junctions (n = 188,543) from WT, *SCAF4 KO* (4KO), *SCAF8 KO* (8KO) and dKO cells. (J) Distance between ALE sites upregulated in dKO (n = 142) and the closest upstream exon-intron junction (observed) compared to distances expected by a random distribution of binding clusters within introns (expected). (K) Distance between IpA sites upregulated in dKO based on 3′Seq and mRNA-Seq (n = 530) and the closest upstream exon-intron junction (observed) compared to distances expected by a random distribution within introns (expected). (L) Strand-specific mNET-Seq metagene profiles for all protein coding genes (n = 16,077) in WT, *SCAF4* KO (4KO), *SCAF8* KO (8KO) and dKO cells. (M) qPCR measurement of mature U1 snRNA levels in WT, *SCAF4* KO (4KO), *SCAF8* KO (8KO) and dKO cells day 5 (d5) day 7 (d7) and d9 (d9) after Dox removal. Delta Ct values are normalized to *GAPDH* levels and shown relative to WT. Error bars indicate ± SD.

**Figure 6:**
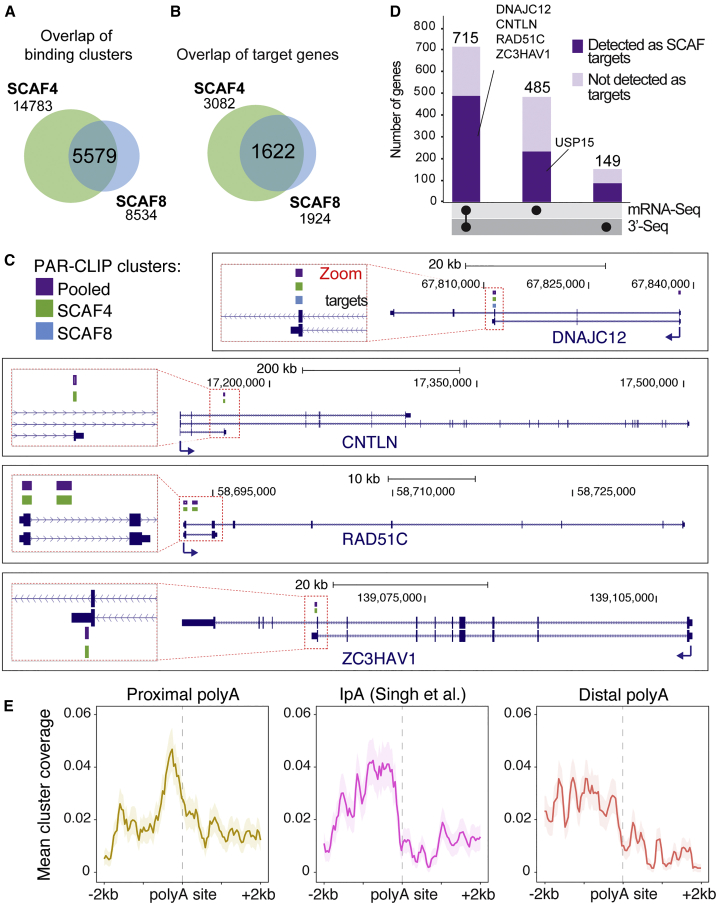
SCAF4 and SCAF8 Bind to Nascent RNA Close to Early polyA Sites (A) Overlap of SCAF4 and SCAF8 consensus PAR-CLIP clusters (found in ≥2 out of 3 biological replicates for both, but not in controls). (B) Overlap of SCAF4 and SCAF8 consensus targets (genes containing ≥2 SCAF4 and SCAF8 consensus clusters). (C) Genome browser examples (zooms on the left) of transcripts with SCAF4 and SCAF8 clusters. (D) Genes with evidence of polyA site changes in the dKO identified from mRNA-seq and/or 3′-seq data. Regulated genes containing SCAF4 and SCAF8 clusters are indicated in dark purple. (E) Mean SCAF4 and SCAF8 CLIP cluster coverage centered around proximal (left panel) and distal (right panel) polyA sites from MISO-regulated genes, or from IpA sites detected by Singh et al. (2018) (middle panel). See also Figure S7 and Table S2.

As a strong indication of the occurrence of the desired, direct nascent RNA-SCAF protein crosslinks, we observed the characteristic T→C transitions in numerous, unique sequence reads of RNA isolated with SCAF4 and SCAF8 (Figure S7A). To obtain high-confidence RNA-binding sites, we initially only considered a stringent set of SCAF binding clusters that were found in 2 out of 3 biological replicates (each supported by >10 unique reads with >8 T→C transitions) in the respective SCAF IP samples, but not in the untagged cell line control or significantly in the input samples (see STAR Methods for details). As expected, most clusters (>95%) were located within annotated transcripts (Figure S7B). Remarkably, among the SCAF8 binding sites (8,534), no less than 65% (5,579) were also SCAF4 binding sites (p value for overlap <1E-100) (Figures 6A and 6B), strongly indicating that the proteins bind the same RNA targets. In further strong support of this conclusion, the correlations of cluster overlaps between SCAF4 and SCAF8 samples were remarkably similar to those observed within the SCAF4 (or SCAF8) triplicates (Figure S7C). In general, more high-confidence SCAF4 sites than SCAF8 sites were uncovered, most likely for technical reasons rather than a biologically significant difference in their binding patterns (see STAR Methods). Because SCAF4 and SCAF8 essentially bound the same sites, we also created a “pooled” set of binding clusters that in addition to the SCAF4 and SCAF8 consensus sites also contained clusters with evidence for either SCAF4 *or* SCAF8 binding, to capture a more complete set of high-confidence SCAF4 and SCAF8 binding sites.

Inspection of binding clusters within individual genes whose termination was affected by *SCAF4 SCAF8* dKO revealed that, remarkably, many SCAF binding clusters were located close to the proximal polyA sites associated with shorter mRNA isoforms (Figure 6C), supporting the idea that SCAF4 and SCAF8 *directly* regulate transcript cleavage at these “early” polyA sites. This was also the case for *DNAJC12* and *ZC3HAV1*, both of which (only) had a SCAF4 and SCAF8 RNA-binding cluster overlapping with the ALE characteristic of their short mRNA isoform, but RNA-binding clusters were generally often detected in genes showing *SCAF*-dependent usage of proximal polyA sites (Figures 6C, 5C, and 5D).

To generally assess the overlap between SCAF RNA-binding sites and genes with alternative polyAsite selection and/or termination in *SCAF4 SCAF8* dKOs, we identified genes with evidence for alternative polyA site selection from either mRNA-seq, 3′-seq, or both. Out of the total of 1,349 genes with such evidence, ∼60% were found to also harbor a SCAF binding site by PAR-CLIP (Figure 6D). This overlap is highly significant (p = 2.8E-67) (Table S2). Given that these genome-wide results were obtained with fundamentally different experimental approaches that are highly unlikely to capture all events, these data provide compelling evidence that the SCAF proteins are indeed present at the genes whose polyA site selection is affected by *SCAF4 SCAF8* dKO. In further support of this, SCAF4 and SCAF8 binding peaked 50–200 nt upstream of proximal polyA sites upregulated in response to loss of SCAF4 and SCAF8 (Figure 6E). This fits nicely with a scenario in which SCAF4 and SCAF8 recognize elements within nascent RNA as it emerges from the RNAPII elongation complex, prior to transcription across the polyA site. Analysis of the CLIP sites revealed several short, low-complexity, C/G-rich RNA-binding motifs, including CG[G/A], [C/G]CAG[C/G], and C[U/A]CC, as overrepresented (Figure S7D). As the relationship between RNA sequence motifs, secondary structures, and the affinities for RNA-binding proteins are often of a complex nature, the precise determinants for SCAF4 and SCAF8 target recognition will need to be addressed in future work.

**Figure 7:**
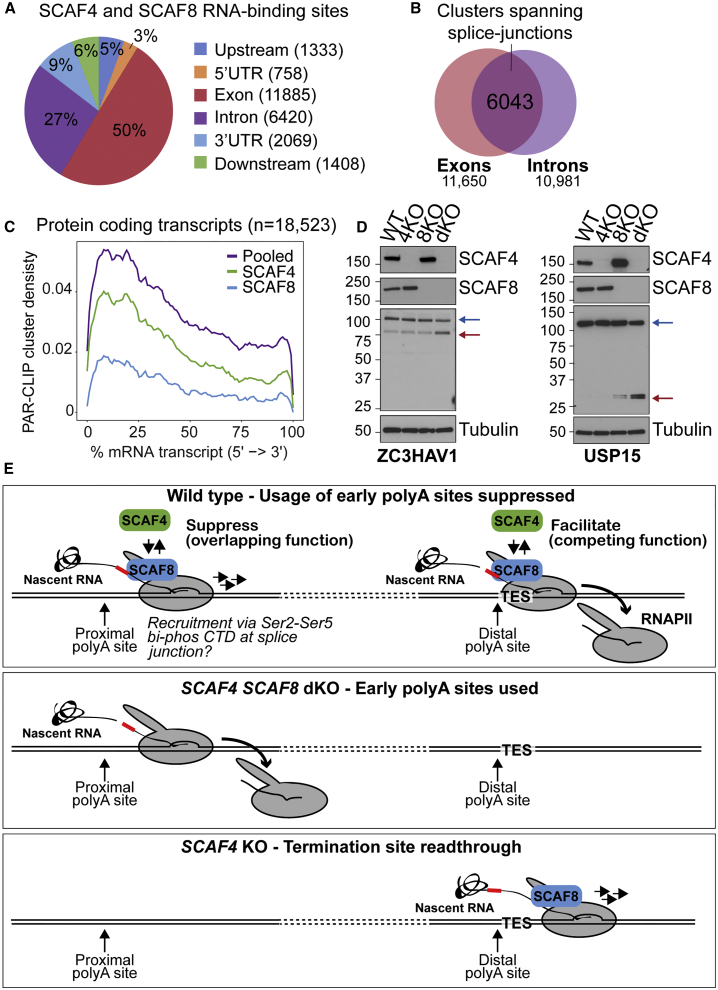
Early polyA Site Selection in *SCAF4* and *SCAF8* dKOs Leads to Production of Truncated Proteins (A) Distribution of pooled SCAF4 and SCAF8 consensus clusters (overlapping clusters found in ≥2 out of 6 samples from SCAF4 and/or SCAF8 experiments, but not in controls). Localization calculated from the cluster midpoint. Upstream, 2 kb upstream of TSS. Downstream, 2 kb downstream of TES. (B) Number of pooled SCAF4 and SCAF8 clusters found exclusively in exons or introns, as well as in clusters spanning an intron-exon (n = 2,271) or exon-intron (n = 3,772) boundary. (C) Meta-transcriptome profiles for SCAF binding clusters across mRNA transcripts. (D) Western blot of protein products produced from the shorter RNA isoforms of *ZC3HAV1* and *USP15* by antibodies raised against N-terminal peptides common to both isoforms. (E) Simple working model and summary of the main effects of SCAF4 and SCAF8 on polyA site selection and transcriptional termination. See also Figure S7.

We also performed metagene analysis to further characterize the SCAF binding clusters. Interestingly, when mapped as the midpoint of clusters, no less than 49% of the binding sites were located in exons, while only 26% were detected in the generally much longer introns (Figure 7A). A similar distribution was observed for SCAF4 or SCAF8 binding clusters when considered separately (Figure S7E). Crucially, a very large number of binding cluster reads spanned splice junctions (Figure 7B), strongly indicating that, as expected, the nuclear SCAF4 and SCAF8 proteins be binding to the nascent RNA *prior* to co-transcriptional mRNA processing, including transcript cleavage. Looking more closely at the RNA-binding pattern around splice junctions, we primarily detected binding on the exon side of the junctions (Figures S7F and S7G). Interestingly, the SCAF binding sites thus overlap with the striking peaks of Ser5P (and Ser2P) RNAPII over the same areas recently detected by mNET sequencing (mNET-seq) (Nojima et al., 2015, Nojima et al., 2018) and are therefore in agreement with the strong preference of both SCAF4 and SCAF8 for binding Ser2-Ser5 double-phosphorylated CTD peptides (Figures 2B, S7F, and S7G). Together, these findings are consistent with the idea that SCAF4 and SCAF8 are recruited to their RNA binding sites at least partly via initial recognition of bi-phosphorylated RNAPII CTD, while, conversely, the data also raised the possibility that the SCAFs might affect RNAPII transcription dynamics at such junctions. Interestingly, however, mNET-seq analysis of *SCAF4 SCAF8* dKO cells indicated no noteworthy changes in RNAPII dynamics at splice junctions, or genome-wide (Figures S7H and S7I), arguing against a role for the SCAFs in the regulation of RNAPII pausing at such junctions, and in agreement with their lack of effect on mRNA splicing.

Further metagene analysis showed that SCAF4 and SCAF8 RNA-binding clusters were generally located throughout transcripts rather than near the TES of genes (Figure 7C), in agreement with a primary role of SCAF4 and SCAF8 in suppressing proximal polyA site usage and premature termination, rather than in 3′ end processing or termination at canonical TESs. Indeed, only 9% and 6% of SCAF4/SCAF8 binding sites were located within 3′ UTRs or downstream of the TES, respectively (Figure 7A). This is in marked contrast to budding yeast Pcf11 and the fission yeast SCAF4/SCAF8 ortholog Seb1, which both display a marked preference for RNA binding downstream of the polyA site (Baejen et al., 2017, Wittmann et al., 2017).

We were initially puzzled by the fact that alternative polyA sites are typically found in introns (hence “intronic polyA” sites or IpA [Singh et al., 2018]), yet the majority of SCAF binding sites were seemingly near splice junctions and at exons. However, this conundrum was explained by the finding that actually IpA sites are *also* generally found near splice junctions (Figures S7J and S7K). Indeed, both among the ALE genes with positive RED score but also among the larger group of IpA sites (Singh et al., 2018) that were utilized in our cell lines, the IpA site was significantly closer to exons than expected by chance (p < 1E-100 for both; Figures S7J and S7K). These data again agree with a model in which SCAF proteins are attracted to the vicinity of early, alternative polyA sites at least partly via recruitment to Ser2-Ser5 phosphorylated RNAPII at splice junctions.

Given that *SCAF4 SCAF8* dKO is lethal, it seems an obvious possibility that the redundant, essential function of SCAF4 and SCAF8 is to act as anti-termination factors that suppress the selection of early polyA sites so that correct mRNA processing and production of functional protein isoforms is ensured. Because the shorter mRNA isoforms detected in dKO cells are spliced and polyadenylated (i.e., stable mRNAs), we investigated whether these shorter mRNA isoforms are indeed translated into detectable proteins. Strikingly, for both the gene and protein examples tested for which suitable antibodies were available, namely, *ZC3HAV1* and *USP15*, the protein products produced from the shorter mRNA isoform were detected in dKO cells (Figure 7D, red arrow). Production of the shorter protein isoform was associated with decreased production of the longer, functional isoform (blue arrow), in agreement with a model in which a general shift toward a less active proteome affects the viability of *SCAF4 SCAF8* dKO cells.

## Discussion

The mechanism, regulation, and factor requirement of polyA site selection and transcriptional termination remain poorly understood. Here, we present evidence for a redundant, essential function for the human RNAPII-interacting proteins SCAF4 and SCAF8 as mRNA anti-terminator proteins, which suppress the use of early, alternative polyA sites to diminish the production of non-functional mRNAs and proteins (Figure 7E). Anti-terminator proteins are well known in bacteriophage and bacteria, and anti-terminator activity has also recently been described for the single-subunit RNA polymerase system in human mitochondria (Santangelo and Artsimovitch, 2011, Posse et al., 2015, Hillen et al., 2017), but, to the best of our knowledge, this is the first report of anti-terminator proteins acting at genes transcribed by eukaryotic, multi-subunit RNA polymerases. Besides their redundant, essential function, SCAF4 and SCAF8 have also evolved distinct, individual functions. SCAF8 is a positive elongation factor, while SCAF4 is also important for termination at the canonical TES of many genes (Figure 7E).

Our knowledge of the function and characteristics of the SCAF4 and SCAF8 proteins has hitherto largely been limited to what is also intrinsic to their protein sequence: these proteins have the hallmarks of RNAPII-interacting proteins that function in RNA biology (Yuryev et al., 1996, Patturajan et al., 1998). Much more is known about the yeast SCAF orthologs, but—intriguingly—the interesting and important data uncovered by work on *S. cerevisiae* Nrd1 and *S. pombe* Seb1 are arguably of somewhat limited predictive value for the key functions uncovered here for human SCAF4 and SCAF8. Indeed, whereas Nrd1 appears to mainly function as part of a Nrd1-Nab3-Sen1 complex to allow termination of non-polyadenylated RNAPII transcripts in cooperation with the nuclear exosome (see, for example, (Arigo et al., 2006, Vasiljeva et al., 2008, Schulz et al., 2013), SCAF4 and SCAF8 do not have stably associated partner proteins, and no evidence for a Nrd1-like function in termination of non-polyadenylated RNAPII transcripts was uncovered. The SCAF homolog in *S. pompe*, Seb1 promotes polyA site selection, so that RNAPII often fails to disassociate downstream of canonical polyA sites in its absence (Lemay et al., 2016, Wittmann et al., 2017). In this aspect, SCAF4 (but not SCAF8) may be somewhat functionally related to Seb1. However, whereas Seb1 recognizes Ser2P RNAPII and primarily associates with RNA at the 3′ end of genes (Baejen et al., 2017, Wittmann et al., 2017), SCAF4 and SCAF8 recognize RNAPII doubly phosphorylated at Ser2 and Ser5 and primarily associate with RNA in the gene body and around splice junctions, befitting their important function in suppressing early polyA site selection and subsequent transcriptional termination. At first glance, it may seem counter-intuitive that SCAF4 can be involved both in termination and anti-termination, but this phenomenon is also observed in bacteria where NusA and NusG sometimes aid termination and sometimes inhibit it (Kuehner et al., 2011, Santangelo and Artsimovitch, 2011).

The co-transcriptional nature of mRNA processing is well established: mRNA capping occurs as the nascent RNA leaves the RNAPII RNA exit channel; splicing is initiated as the polymerase transcribes across the junctions between exons and introns; and transcript cleavage at polyadenylation sites is coupled to the transcribing RNAPII as well (Bentley, 2014, Proudfoot, 2016). In the present study, we found that, even though *SCAF8* KO cells have a low transcript elongation rate, there is no noteworthy increase in exon inclusion or indeed any marked effect on cassette exon splicing in these cells. The overall effect on mRNA splicing in *SCAF4* KO, *SCAF8* KO, and the *SCAF4 SCAF8* dKO is thus remarkable only by its relative absence.

In contrast, the *SCAF4 SCAF8* dKO cells in particular display a dramatic change in early polyA site selection and downstream transcriptional termination, resulting in short mRNA isoforms with ALEs. Importantly, this effect is not equivalent to that of traditional termination factors, such as *PCF11* whose mutation primarily results in polyA sites at the end of genes being ignored (West and Proudfoot, 2008, Kamieniarz-Gdula et al., 2019). Instead, *SCAF4 SCAF8* dKO results in increased use of early, gene-intrinsic, alternative polyA sites, which results in premature transcript termination. The intriguing role for SCAF4 and SCAF8 as mRNA anti-terminators raises questions about the underlying molecular mechanism. Although the process of transcript termination differs between prokaryotes and eukaryotes (see Introduction), it is relevant to note that previously described prokaryotic anti-terminators fall into two classes: (1) site-specific anti-terminators that work through recognition of *cis*-regulatory RNA elements, and (2) anti-terminators that act via an ability to increase RNAP processivity, such as the bacterial anti-terminator RfaH (Santangelo and Artsimovitch, 2011, Kang et al., 2018). The precise, molecular mechanism underlying the function of SCAF4 and SCAF8 is unknown, and understanding it is a key, future goal. However, as outlined in further detail below, we speculate that SCAF4 and SCAF8 direct polyA site selection at least partly by detecting *cis*-regulatory RNA elements in the nascent RNA emerging from the transcribing polymerase. While an effect of SCAF proteins on RNAPII processivity cannot be ruled out, it appears less likely that suppression of early polyA site usage is an indirect effect of altered transcript elongation rates, for example. Indeed, we uncovered no persuasive correlation between early polyA site selection and elongation rates. Elongation rates are thus decreased in both *SCAF8* single KO and dKO cells (but not in *SCAF4* single KO), yet the shift toward usage of early polyA sites is much stronger in the dKO (and even in the *SCAF4* single KO) than they are in the *SCAF8* single KO. Although any factor affecting transcript elongation might be expected to have *some* effect on the choice of polyA and transcription termination sites, the effect of SCAF4 and SCAF8 is unique in its extent and specificity: *SCAF4 SCAF8* dKO affects premature polyA site selection and termination in more than 1,300 genes and at specific sites, most of which have a nearby SCAF binding site.

It is worth noting that the changes in polyA site selection and termination observed in the *SCAF4 SCAF8* dKO are distinct from those caused by “telescripting” (Oh et al., 2017 and references cited therein). In telescripting, which occurs when U1 snRNA is depleted, most pre-mRNAs are terminated downstream of cryptic, intronic polyadenylation signals that are typically within a short distance (∼1 kb) of the TSS. In contrast, SCAF4 and SCAF8 do not generally suppress cryptic polyA sites and thus have little effect on global RNAPII transcription dynamics (Figure S7L), but rather suppress the usage of a number of specific polyA sites that, when utilized, can produce correctly processed, stable transcripts that may be translated into proteins. *SCAF4 SCAF8* dKO has little or no effect on U1 snRNA levels (Figure S7M), further ruling out an indirect effect via the telescripting pathway.

Together, our data support a working model in which SCAF4 and SCAF8 are suppressors of gene-intrinsic polyA site usage, initially by recognition of the “CTD status” of RNAPII and subsequently by binding RNA sequence elements within the nascent transcript, to allow premature polyA sites to be ignored (Figure 7E). Intriguingly, SCAF4 and SCAF8 preferentially bind CTD repeats containing doubly phosphorylated Ser2P-Ser5, an unusual mark that appears to be particularly enriched on RNAPII complexes briefly stalled at, or passing, splice junctions (Nojima et al., 2015, Nojima et al., 2018). We propose that such RNAPII complexes in effect serve to initially enrich SCAFs near the early polyA sites whose usage they suppress. It may seem counterintuitive that SCAF4 and SCAF8 cannot associate with the same RNAPII complex when they recognize a phosphorylation signature, which could, in principle, occur many times in the same CTD tail. However, as previously shown for the Mediator complex (Robinson et al., 2016), the CTD should merely be viewed as a “landing pad,” which then seeds individual weak interactions with the body of RNAPII. Indeed, recent data show that the SCAF4/8 ortholog Seb1 interacts not only with the CTD but also with the body of RNAPII near the RNA exit channel (Kecman et al., 2018). Given that SCAF4 and SCAF8 are highly similar proteins, it thus seems likely that they contact the same binding sites on the body of RNAPII, thus rendering polymerase association mutually exclusive. Once associated with RNAPII near splice junction, the SCAFs would be perfectly placed to detect signals in the nascent RNA emerging from the transcribing polymerase. Gratifyingly, the SCAF binding sites we uncovered are positioned 50–200 nt upstream of early polyA sites, opening the possibility that SCAF are deposited at such binding sites in advance of the emergence from RNAPII of the polyA sites they regulate. It is tempting to further speculate that regulation might be brought about directly or indirectly through an effect on the CPSF complex, with which the SCAFs interact. The precise nature of the putative RNA elements recognized by the SCAFs remains to be determined, but we note that sequence-directed RNA recognition by the yeast SCAF orthologs Nrd1 and Seb1 via their RNA recognition motif (RRM) has previously been reported (Steinmetz and Brow, 1996, Carroll et al., 2004, Lemay et al., 2016, Wittmann et al., 2017), providing precedence and a conceptual framework for further investigation.

Transcriptome complexity has greatly increased during evolution and more than 90% of human genes encode multiple transcript isoforms (Wang et al., 2008). Alternative transcript isoforms, including short transcript isoforms with alternative last exons (ALE isoforms), thus provide an opportunity to fine-tune the transcriptional output in a cell- or tissue-specific manner, during development or in response to stimuli. However, it is evident that evolution of such isoforms must come with an increased need for suppressing their untimely, general expression in cells. It is thus tempting to speculate that the gene duplication that gave rise to *SCAF4* and *SCAF8*, combined with the longer, more complex, intron- and polyA site-rich genes typical of vertebrates, has both allowed and necessitated the divergent evolution of *SCAF4* and *SCAF8* to specifically deal with the challenges posed by gene-intrinsic transcript termination sites.

## STAR★Methods

### Key Resources Table

**Table:** 

REAGENT or RESOURCE	SOURCE	IDENTIFIER
**Antibodies**		

Polyclonal to CPSF73/CPSF3	Bethyl	A301-090A, RRID: AB_873009
Polyclonal to CPSF100/CPSF2	Bethyl	A301-583A, RRID: AB_1078866
Polyclonal to CPSF160/CPSF1	Bethyl	A301-580A, RRID: AB_1078859
Polyclonal to CTR9	Bethyl	A301-395A, RRID: AB_960973
Monoclonal to FLAG	Sigma	F1804, RRID: AB_262044
Polyclonal to LEO1	Bethyl	A300-174A, RRID: AB_309451
Polyclonal to PAF1	Bethyl	A300-172A, RRID: AB_309394
Polyclonal to RECQL5	Abcam	ab91422, RRID: AB_2050245
Polyclonal to total RNAPII (N-20)	Santa Cruz	sc-899, RRID: AB_632359
Monoclonal to RNAPII total CTD	MBL International	MABI0601, RRID: AB_2728735
Monoclonal to RNAPII phosphorylated CTD (4H8)	Cell services, The Francis Crick Institute	N/A
Polyclonal to total RNAPII (D8L4Y)	Cell Signaling	14958, RRID: AB_2687876
Monoclonal to total RNAPII (ARNA-3)	Sigma	CBL221, RRID: AB_2167489
Monoclonal to Ser2P RNAPII (3E10)	Kind gift from Dirk Eick	N/A
Monoclonal to Ser5P RNAPII (3E8)	Kind gift from Dirk Eick	N/A
Monoclonal to Ser7P RNAPII (4E12)	Kind gift from Dirk Eick	N/A
Monoclonal to Thr4P RNAPII (6D7)	Kind gift from Dirk Eick	N/A
Monoclonal to Tyr1P RNAPII (3D12)	Kind gift from Dirk Eick	N/A
Polyclonal to RPRD1A	Atlas Antibodies	HPA040602, RRID: AB_10673137
Polyclonal to SCAF4	Bethyl	A303-951A, RRID: AB_2620300
Polyclonal to SCAF8/RBM16	Bethyl	A301-037A, RRID: AB_2253436
Polyclonal to SUPT6H	Bethyl	A300-801A, RRID: AB_577215
Monoclonal to Tubulin	Sigma	T5168, RRID: AB_477579
Monoclonal to USP15, clone 1C10	Novusbio	H00009958-M01, RRID: AB_2257149
Monoclonal to Vinculin	Sigma	V9131, RRID: AB_477629
Polyclonal to WRD33	Bethyl	A301-152A, RRID: AB_2215378
Polyclonal to ZC3HAV1/ZAP	ProteinTech	16820-1-AP, RRID: AB_2728733
Anti-mouse HRP	Santa Cruz	sc-516102, RRID: AB_2687626
Anti-rabbit HRP	Jackson ImmunoResearch	711-035-152, RRID: AB_10015282
Anti-rat HRP	Jackson ImmunoResearch	112-035-003, RRID: AB_2338128

**Bacterial and Virus Strains**		

NEB 5-alpha Competent *E. coli*	NEB	C2988J
One Shot ccdB Survival 2 T1R Competent Cells	Thermo Fisher Scientific	A10460

**Chemicals, Peptides, and Recombinant Proteins**		

Doxycycline	Clontech	8634-1
MG132	Cayman Chemical	10012628
N-Ethylmaleimide (NEM)	Sigma-Aldrich	E3876
4-thiouridine	Glentham Life Sciences	GN6085
4-thiouracil	Sigma-Aldrich	440736
DRB (5,6-dichloro-1-β-D-ribofuranosylbenzimidazole)	Sigma-Aldrich	D1916
MTSEA biotin-XX linker ((MTSEA Biotincapcap; 2-((6-((6-((biotinoyl)amino)hexanoyl)amino)hexanoyl)amino)ethylmethanethiosulfonate))	Biotium	BT90066
3xFLAG peptide	Peptide Chemistry, The Francis Crick Institute	N/A
BioCTD28-non phosphorylated peptide	Peptide Chemistry, The Francis Crick Institute	N/A
BioCTD28-Tyr1P peptide	Peptide Chemistry, The Francis Crick Institute	N/A
BioCTD28-Ser2P peptide	Peptide Chemistry, The Francis Crick Institute	N/A
BioCTD28-Thr4P peptide	Peptide Chemistry, The Francis Crick Institute	N/A
BioCTD28-Ser5P peptide	Peptide Chemistry, The Francis Crick Institute	N/A
BioCTD28-Ser2P+Ser5P peptide	Peptide Chemistry, The Francis Crick Institute	N/A
BioCTD28-Ser2P+Tyr1P peptide	Peptide Chemistry, The Francis Crick Institute	N/A

**Critical Commercial Assays**		

RNeasy kit	QIAGEN	74104
miRNeasy kit	QIAGEN	217004
RNA minElute clean-up kit	QIAGEN	74204
RNase-Free DNase Set	QIAGEN	79254
PureLink RNA Mini kit	Thermo Fisher Scientific	12183020
μMACS Streptavidin Kit	Miltenyi	130-074-101
Taqman Reverse Transcriptase Reagents	Thermo Fisher Scientific	N8080234
SilverQuest Silver Staining Kit	Thermo Fisher Scientific	LC6070
NEBNext Multiplex Small RNA Library Prep Set for Illumina (Set 1)	NEB	E7300S
NEBNext Multiplex Small RNA Library Prep Set for Illumina (Set 2)	NEB	E7580S
NuGEN ultra low V2 kit	NuGEN	0344
TruSeq HT kit	Illumina	20020595
Strand-specific TruSeq total RNA kit	Illumina	20020597

**Deposited Data**		

Sequencing data	This study	GSE60358

**Experimental Models: Cell Lines**		

Flp-In T-Rex HEK293 cells	Thermo Fisher Scientific	R78007
Flp-In T-Rex HEK293 4KO/TO/GFP-SCAF4	This study	N/A
Flp-In T-Rex HEK293 dKO1/TO/GFP-SCAF4	This study	N/A
Flp-In T-Rex HEK293 dKO2/TO/GFP-SCAF4	This study	N/A
Flp-In T-Rex HEK293 8KO/TO/GFP-SCAF8	This study	N/A
Flp-In T-Rex HEK293 dKO3/TO/GFP-SCAF8	This study	N/A
Flp-In T-Rex HEK293 dKO4/TO/GFP-SCAF8	This study	N/A
Flp-In T-Rex HEK293 TO/FLAG-SCAF4	This study	N/A
Flp-In T-Rex HEK293 TO/FLAG/HA-SCAF8	This study	N/A

**Experimental Models: Organisms/Strains**		

*S. cerevisiae* (strain BY4741, MATa, his3D1, leu2D0, met15D0, ura3D0)	Euroscarf	BY4741(Y00000)

**Oligonucleotides**		

All oligonucleotides used in this study are listed in Table S3	This paper	N/A

**Recombinant DNA**		

For all plasmids generated within this study see Table S3	This study	N/A
pDONR223	Kind gift from Simon Boulton	N/A
pENTR4 dual selection	Thermo Fisher Scientific	A10465
pFRT/TO/GFP DEST	Kind gift from Markus Landthaler	N/A
pFRT/TO/FLAGHA DEST	Kind gift from Markus Landthaler	N/A
pFRT/TO	Kind gift from Markus Landthaler	N/A
pOG44	Thermo Fisher Scientific	V600520
pX461	Addgene	48140
pOTB7 SCAF4	SourceBioscience	IRAUp969D03110D, IMAGE ID 5432277/LLCM1905 M22
pBluescriptR SCAF8	SourceBioscience	IRATp970F1287D, IMAGE ID 4374384/AT87 F12

**Software and Algorithms**		

Cytoscape version 3.6.1	Su et al., 2014	https://www.cytoscape.org/download.php
MISO	Katz et al., 2010	https://genes.mit.edu/burgelab/miso/
MaxQuant version 1.3.05	Tyanova et al., 2016	https://www.maxquant.org
Perseus version 1.4.0.11	Tyanova et al., 2016	http://maxquant.net/perseus/
MEGA version 6.06	Tamura et al., 2013	https://www.megasoftware.net/
STAR version 2.3.0	Dobin et al., 2013	https://github.com/alexdobin/STAR
SAMtools	Li and Durbin, 2009	http://www.htslib.org/
Bowtie version 2.2.3	Langmead and Salzberg, 2012	https://sourceforge.net/projects/bowtie-bio/files/bowtie2/2.2.3/
BEDtools	Quinlan and Hall, 2010	https://bedtools.readthedocs.io/en/latest/
PARalyzer	Corcoran et al., 2011	https://ohlerlab.mdc-berlin.de/software/PARalyzer_85/
Ngs.plot	Shen et al., 2014	https://github.com/shenlab-sinai/ngsplot
Cutadapt	Martin 2011	https://cutadapt.readthedocs.io/en/stable/index.html
RSEM	Li and Dewey, 2011	https://github.com/deweylab/RSEM
MEME-ChIP	Machanick and Bailey 2011	http://meme-suite.org/tools/meme-chip

**Other**		

High glucose DMEM	Thermo Fisher Scientific	11965118
Tet-free FBS	Clontech	631106
Poly-lysine	Sigma-Aldrich	P7280
VECTASHIELD Antifade Mounting Medium containing DAPI	Vector Laboratories	H-1200
3-8% Tris-Acetate gels	BioRad	3450130
4-15% TGX gels (18wells/26/wells)	BioRad	56711084/5
Complete EDTA-free protease inhibitor cocktail	Sigma-Aldrich	05056489001
PhosSTOP	Sigma-Aldrich	04906837001
Nitrocellulose membrane	GE Healthcare Life Sciences	10600002
SuperSignal West Pico PLUS ECl reagent	Thermo Fisher Scientific	34577
SuperSignal West Dura ECl reagent	Thermo Fisher Scientific	34075
Pierce Spin Columns	Thermo Fisher Scientific	69705
Protein G agarose beads	Sigma-Aldrich	11719416001
InstantBlue	Expedeon	ISB1L
Micro Bio-Spin P-30 Gel Columns	BioRad	7326223
iTaqUniversal SYBR Green Supermix	BioRad	172-5124
ANTI-FLAG M2 Affinity Gel	Sigma-Aldrich	A2220
Benzonase	MerckMillipore	70746-4
Gateway LR Clonase II Enzyme	Thermo Fisher Scientific	11791020
Lipofectamine 2000	Thermo Fisher Scientific	11668019
M-280 Streptavidin dynabeads	Thermo Fisher Scientific	11205D
RNaseI	Thermo Fisher Scientific	AM2294
TURBO DNase	Thermo Fisher Scientific	AM2238
ProtinaseK	Sigma-Aldrich	3115887001
AMPureXP beads	Beckman Coulter	A63881
T4 Polynucleotide Kinase	Thermo Fisher Scientific	EK003
TRIzol Reagent	Thermo Fisher Scientific	15596026

### Contact for Reagent and Resource Sharing

Further information and requests for resources and reagents should be directed to and will be fulfilled by the Lead Contact, Jesper Q. Svejstrup (jesper.svejstrup@crick.ac.uk). Plasmids were deposited with and will be distributed through the non-profit distributor Addgene.

### Experimental Model and Subject Details

#### Cell lines and culture conditions

Flp-In T-REx HEK293 cells (Thermo Fisher Scientific, R78007, human embryonic kidney epithelial, female origin) were cultured in high glucose DMEM (Thermo Fisher Scientific, 11965118) supplemented with 10% v/v FBS, 100 U/mL penicillin, 100 μg/mL streptomycin, 2 mM L-glutamine, 100 μg/mL zeocin and 15 μg/mL blasticidin at 37°C with 5% CO2 and routinely passaged 2-3 times a week. All cell lines were confirmed to be mycoplasma-free.

### Method Details

#### Plasmid construction

The coding region of SCAF4 and SCAF8 were amplified from ORF clones (SourceBioscience, Key Resources Table) with primers adding attB1 and attB2 recombination sites (Table S3) and recombined into pDONR223 using the gateway BP recombinase system (Thermo Fisher Scientific, 11789020). To generate a CRISPR resistant SCAF4 construct, a DNA fragment containing synonymous substitutions in regions recognized by the guide RNAs (Table S3) was subcloned into pDONR233 SCAF4 using BamHI/XmnI sites. To generate CRISPR resistant SCAF8 constructs corresponding to isoform C (GenBank: NP_001273123.1), two DNA fragments containing synonymous substitutions in regions recognized by the two guide RNAs (Table S3) were consecutively subcloned into pDONR233 SCAF8 using first AfeI/BstBI and then MscI/Sac sites. To generate CRISPR resistant SCAF8 constructs corresponding to isoform D that utilizes a downstream in frame start codon (GenBank: NP_001273128.1), a DNA fragment containing synonymous substitutions in regions recognized by the two guide RNAs (Table S3) was subcloned into pDONR233 SCAF8 using MscI/Sac sites. All DNA fragments were synthesized and sequence-verified by GenScript. pDONR223 constructs were recombined into the pFRT/TO/FLAG/HA-DEST or pFRT/TO/GFP-DEST destination vector using Gateway LR Clonase II Enzyme mix according to the manufacturer’s protocol (Thermo Fisher Scientific, 11791020). FLAG-tagged SCAF4 was amplified using primers listed in Table S3 and cloned into pFRT/TO using KnpI/NotI sites.

#### Generation of stable cell lines

Flp-In T-REx HEK293 cell lines expressing inducible GFP-tagged SCAF4 or SCAF8 were generated as described previously (Gregersen et al., 2014). Briefly, Flp-In T-REx HEK293 cell lines maintained in 100 μg/mL zeocin and 15 μg/mL blasticidin prior to transfection, were co-transfected with a 9:1 ratio of pOG44 Flp-recombinase expression vector (Thermo Fisher Scientific, V600520) and pFRT/TO/GFP-SCAF4 or pFRT/TO/GFP-SCAF8 CRISPR resistant constructs using Lipofectamine 2000 (Thermo Fisher Scientific, 11668019) according to the manufacturer’s instructions. 24 h after transfection, cells were seeded as single cells and after another 24 h the cell culture media was supplemented with 100 μg/mL hygromycin and 15 μg/mL blasticidin. Expression of GFP-tagged proteins was induced overnight by the addition of doxycycline (Clontech, 8634-1, 1 μg/mL final concentration) and verified by western blotting using antibodies against SCAF4 or SCAF8. CRISPR-Cas9-nickase-mediated genome editing of Flp-In T-REx HEK293 GFP-SCAF4/GFP-SCAF8 cell lines was performed as previously described (Ran et al., 2013). The oligonucleotides encoding sgRNAs for targeting the coding region of SCAF4 or SCAF8 are listed in Table S3. Briefly, the forward and reverse strand oligonucleotides were annealed and ligated into pSpCas9n(BB)-2A-GFP (Addgene, PX461) linearized with BbsI, and plasmids were sequenced after cloning and transformation. To generate knockouts, cells were co-transfected with the two pSpCas9n(BB)-2A-GFP plasmids containing nickase gRNA pairs A and B using Lipofectamine 2000 (Thermo Fisher Scientific, 11668019) according to the manufacturer’s instructions. 48 h after transfection, high GFP positive cells (with GFP levels higher than untransfected GFP-SCAF4 or GFP-SCAF8 expressing cells) were sorted clonally by FACS into 96-well plates and cultivated until colonies were obtained, and cells lacking endogenous SCAF4 or SCAF8 selected. To follow decay of GFP-tagged rescue proteins, cells were washed 3 times in PBS and seeded into tetracycline-free selection media (high glucose DMEM (Thermo Fisher Scientific, 11965118) supplemented with 10% v/v tet-free FBS (Clontech, 631106), 100 U/mL penicillin, 100 μg/mL streptomycin, 2 mM L-glutamine, 100 μg/mL hygromycin and 15 μg/mL blasticidin). Flp-In T-REx HEK293 cell lines inducible expressing FLAG-tagged SCAF4 or SCAF8 were generated as described above by co-transfection of pOG44 with pFRT/TO/SCAF4-FLAG or pFRT/TO/FLAGHA-SCAF8 and single clones tested for expression of FLAG-tagged SCAF4 or SCAF8 by western blotting.

#### Clonogenic survival assay

Cells stably expressing Dox-inducible GFP-SCAF4 or SCAF8, or CRISPR KOs cells containing GFP-SCAF4 or SCAF8 rescue constructs, were grown in the absence or presence of Dox for 5 days after which 200 cells/well were seeded into 6-well plates in ± Dox containing media. Colonies were fixed by 4% (v/v) formaldehyde 11 days after seeding and stained with a 0.1% (w/v) crystal violet solution. Colonies from two biological replicates (each seeded into triplicate wells) were counted.

#### Growth curves

Cells stably expressing Dox-inducible GFP-SCAF4 or SCAF8, or CRISPR KOs cells containing GFP-SCA4 or SCAF8 rescue constructs, were grown in the absence or presence of Dox for 5 days after which 5000 cells/well were seeded into 24-well plates. Cells were fixed by 4% (v/v) formaldehyde for the following 5 days and stained with 0.1% (w/v) crystal violet solution for 15 min followed by several washes in water. The crystal violet was extracted from four replicate wells using 10% (v/v) acetic acid and absorbance measured at 620 nm and normalized to the first time point (the day after seeding).

#### CID alignment and phylogenetic study

The CID of SCAF4 (GenBank: NP_001138916), SCAF8 (GenBank: NP_001273128), Nrd1 (GenBank: NP_014148), Seb1 (GenBank: NP_593148), Pcf11 (GenBank: NP_010514) and PCF11 (GenBank: NP_056969) were aligned using T-COFFEE (Version_11.00.d625267) and visualized using Jalview 2. A phylogenetic tree (Neighbor joining) was generated using MEGA 6.06 using the Poisson model (Tamura et al., 2013). Bootstrap values were calculated based on 1000 permutations using pairwise deletion of gaps.

#### Microscopy

GFP-SCAF4 or SCAF8 expressing cells were seeded onto poly-lysine (Sigma-Aldrich, P7280) coated coverslips in Dox-containing media. Cells were fixed using 4% (v/v) formaldehyde in PBS for 15 min and mounted onto slides using VECTASHIELD Antifade Mounting Medium containing DAPI (Vector Laboratories, H-1200) and visualized using an inverted SP5 confocal microscope (Leica).

#### Western blotting

For whole cell extracts, cells pellets were lysed in NP-40 lysis buffer (50 mM Tris-HCl pH 7.5, 500 mM NaCl, 2 mM EDTA, 0.5% (v/v) NP-40, 0.5 mM DTT, PhosSTOP (Sigma-Aldrich, 04906837001) and Protease Inhibitor Cocktail (Sigma-Aldrich, 05056489001). 50 μg protein/lane was separated on 3%–8% Tris-Acetate (BioRad, 3450130) or 4%–15% TGX gels (BioRad, 56711084/5) and transferred to nitrocellulose membranes (GE Healthcare Life Sciences, 10600002). Membranes were blocked in 5% (w/v) skimmed milk in PBS-T (PBS, 0.1% (v/v) Tween20) for 1 h at room temperature and incubated with primary antibody (in 5% (w/v) skimmed milk in PBS-T) overnight at 4°C. Primary antibodies are listed in Key Resources Table. Antibody against tubulin or vinculin served as loading controls. Membranes were washed several times in PBS-T, incubated with HRP-conjugated secondary antibody (Key Resources Table) in 5% (w/v) skimmed milk in PBS-T and visualized using SuperSignal West Pico PLUS or Dura Chemiluminescent Substrate ECL reagent (Thermo Fisher Scientific, 34577 or 34075).

#### Quantitative PCR (qPCR)

Total RNA was extracted using the RNeasy kit (QIAGEN, 74104) for nascent and mature RNA, or the miRNeasy kit (QIAGEN, 217004) for snRNA, following the instructions of the manufacturer including an on-column DNase treatment (QIAGEN, 79254). Reverse transcription was performed using TaqMan Reverse Transcription Reagents (Thermo Fisher Scientific, N8080234). For detection of readthrough transcripts, random hexamers were used for the reverse transcription step; for mature mRNA, oligo dT primers were used; and for detection of mature U1 snRNA, gene-specific primers were used. cDNA was amplified using iTaq Universal SYBR Green Supermix (BioRad, 172-5124) with 30 cycles of 15 s denaturation at 94°C, 15 s annealing at 60°C, and 20 s extensions at 72°C. Primers amplifying mature *GAPDH* were used as normalization control. Primer sequences are listed in Table S3. U1 snRNA qPCR primers were published previously (O’Reilly et al., 2013).

#### FLAG-SCAF4 and FLAG-SCAF8 immunoprecipitations

Flp-In T-REx HEK293 cells stably expressing Dox-inducible FLAG-SCAF4 or FLAG-SCAF8 were induced overnight by the addition of Dox (1 μg/mL final concentration). For mass-spectrometry experiments, cells were treated with 5 μM final concentration of the proteasome inhibitor MG132 (Cayman Chemical, 10012628) for 4 h prior to harvest. Immunoprecipitations for western blot validation were done without MG132. Cells were harvested by scraping in ice-cold PBS, washed once in cold PBS and pelleted by centrifugation at 1,500 rpm for 5 min at 4°C. Cells were then fractionated to obtain a soluble fraction (containing cytosolic and nucleoplasmic proteins) and a chromatin fraction. Phosphatase inhibitors (PhosSTOP, Sigma-Aldrich, 04906837001) and Protease Inhibitor Cocktail (Sigma-Aldrich, 05056489001) were added fresh to all buffers. First, cells were resuspended in 2 pellet volumes of hypotonic buffer (10 mM HEPES pH 7.5, 10 mM KCl, 1.5 mM MgCl_2_, 20 mM NEM (N-ethylmaleimide, Sigma-Aldrich, E3876), incubated on ice for 15 min and homogenized with 20 strokes using a loose pestle. Nuclei were pelleted at 3,900 rpm for 15 min and supernatant collected as cytoplasmic fraction. The remaining pellet was resuspended in 2 pellet volumes (original cell pellet volumes) nucleoplasmic extraction buffer (20 mM HEPES pH 7.9, 1.5 mM MgCl_2_, 150 mM potassium acetate, 10% (v/v) glycerol and 0.05% (v/v) NP-40), incubated on ice for 20 min and cleared by centrifugation at 20,000 g for 20 min at 4°C. Supernatant was collected as nucleoplasmic fraction. After correcting the cytoplasmic fractions to 10% (v/v) glycerol, 3 mM EDTA, 0.05% (v/v) NP-40 and 150 mM NaCl final concentration, the cytoplasmic and nucleoplasmic fraction were pooled to obtain a combined soluble fraction. The remaining pellet was resuspended in chromatin digestion buffer (20 mM HEPES pH 7.9, 1.5 mM MgCl_2_, 10% (v/v) glycerol, 150 mM NaCl, 0.1% (v/v) NP-40 and 250 U/mL benzonase [MerckMillipore, 70746-4]) and incubated for 1 h at 4°C. Benzonase digested samples were centrifuged at 20,000 g for 20 min at 4°C and supernatant collected as low salt chromatin fraction. The remaining pellet was resuspended in high salt chromatin extraction buffer (20 mM HEPES pH 7.9, 3 mM EDTA, 1.5 mM MgCl_2_, 10% (v/v) glycerol, 500 mM NaCl and 0.1% (v/v) NP-40) and incubated for 20 min on ice. Prior to centrifugation at 20,000 g for 20 min at 4°C, the salt concentration was diluted back to 150 mM NaCl by addition of high salt dilution buffer (20 mM HEPES pH 7.9, 3 mM EDTA, 1.5 mM MgCl_2_, 10% (v/v) glycerol, 500 mM NaCl and 0.1% (v/v) NP-40). Collected supernatant was pooled with the low salt chromatin fraction to obtain one combined chromatin fraction. For FLAG immunoprecipitations soluble and/or chromatin extracts were incubated with ANTI-FLAG M2 Affinity Gel (Sigma-Aldrich, A2220) at 4°C for 3 h. Beads were washed 5 times in IP wash buffer (150 mM NaCl, 20 mM Tris-HCl pH 7.5, 1.5 mM MgCl_2_, 3mM EDTA, 10% (v/v) glycerol, 0.1% (v/v) NP-40, phosphatase inhibitors (PhosSTOP, Sigma-Aldrich, 04906837001) and protease inhibitor cocktail [Sigma-Aldrich, 05056489001]) with the last wash being on a spin column (Thermo Fisher Scientific, 69705). Immunoprecipitates were eluted using 1 mg/mL 3xFLAG peptide dissolved in IP wash buffer by incubation for 1 h at 4°C. FLAG elutions were run on an SDS-PAGE and stained using the SilverQuest Silver Staining Kit (Thermo Fisher Scientific, LC6070) to confirm immunoprecipitation of full-length SCAF4 and SCAF8. For mass-spectrometry of FLAG immunoprecipitates, eluted proteins were subjected to SDS-PAGE and migrated approximately 1-2 cm into the gel. Proteins were in-gel digested with trypsin, using a Janus Automated Workstation (Perkin Elmer), and peptides were analyzed using an LTQ Orbitrap-Velos mass spectrometer coupled to an Ultimate3000 HPLC equipped with an EASY-Spray nanosource (Thermo Fisher Scientific). Raw data was processed using MaxQuant v1.3.05 (Tyanova et al., 2016). Due to several identical peptides between SCAF4 and SCAF8, the MaxQuant analysis was done separately for the SCAF4 and SCAF8 immunoprecipitates to avoid wrongly assigning common peptides, which would otherwise assign common peptides to the protein with the highest overall peptide count. The proteingroup.txt output table was imported into Perseus software v1.4.0.11 (Tyanova et al., 2016) for further statistical processing, and visualization. Statistical parameters for volcano plots were calculated using two-sided t test for data from two biological replicates (each containing information form triplicate injections). t test difference were plotted against -log10 t test p values. Protein-protein interaction networks were visualized using Cytoscape (version 3.6.1) (Su et al., 2014). The width of the line is representative of the average significance for a given interaction partner while the width of the edge around the node is representative of the average t test difference.

#### Peptide binding assays

Peptide binding assays were performed with biotinylated 4 heptad repeat CTD peptides (28mers) (see Table S3 for peptide sequences) and purified FLAG-tagged SCAF4 and SCAF8 from chromatin enriched fractions from Flp-In T-REx HEK293 cells stably expressing FLAG-SCAF4 or FLAG-SCAF8. Extracts were pre-cleared by incubation with Protein G agarose beads (Sigma-Aldrich, 11719416001) prior to FLAG IP as described above. Purification of FLAG-SCAF4 and SCAF8 was confirmed by silver stain as described above, or by Coomassie stain (InstantBlue, Expedeon, ISB1L). 40 μg of peptides dissolved in PBS was bound to 100 μL of M-280 Streptavidin dynabeads (Thermo Fisher Scientific, 11205D) for 30 min at room temperature, washed twice in PBS-T, once in peptide binding buffer (50 mM NaCl, 10 mM Tris-HCl pH 7.5, 3 mM EDTA, 1.5 mM MgCl_2_, 0.05% NP-40, 1% BSA, Phosphatase inhibitors [PhosSTOP Sigma-Aldrich, 04906837001] and Protease Inhibitor Cocktail [Sigma-Aldrich, 05056489001]). Beads were then incubated with purified FLAG-SCAF4 and FLAG-SCAF8 from chromatin extracts for 2 hr at 4°C on a rotor. Beads were washed twice in peptide binding buffer followed by subsequent washes in increasing salt concentration (200 mM, 400 mM, 800 mM and 1 M NaCl). After each wash 1/5 of resuspended beads were removed and protein eluted by boiling in SDS-containing loading buffer and analyzed by SDS-PAGE.

#### TT-Seq (nascent RNA-Seq) and DRB/TT-Seq

Single and double SCAF4 and/or SCAF8 CRIPSR KO were grown for 5 days with and without Dox prior to *in vivo* labeling of nascent RNA by a 1 mM 4SU (Glentham Life Sciences, GN6085) pulse for 15 min. Labeling was stopped by TRIzol (Thermo Fisher Scientific, 15596026) and RNA extracted accordingly to the manufacturer’s instructions. As a control for equal sample preparation, we spiked-in *S. cerevisiae* (strain BY4741, MATa, his3D1, leu2D0, met15D0, ura3D0) 4-thiouracil (4TU)-labeled RNA. *S. cerevisiae* were grown in YPD medium overnight, diluted to an OD600 of 0.1, and grown to mid-log phase (OD600 of 0.8) and labeled with 5 mM 4TU (Sigma-Aldrich, 440736) for 6 min. Total RNA was extracted using the PureLink RNA Mini kit (Thermo Fisher Scientific, 12183020) following the enzymatic protocol. For purification of 4SU labeled RNA, 100 μg mammalian 4SU labeled RNA was spiked-in with 1/100 of 4TU-labeled *S. cerevisiae* RNA. The 101 μg RNA (in a total volume of 100 uL) was fragmented by addition of 20 μL 1 M NaOH and left on ice for 20 min to obtain RNA fragments between 200-500 nt. Fragmentation was stopped by addition of 80 μL 1 M Tris pH 6.8 and cleaned up twice with Micro Bio-Spin P-30 Gel Columns (BioRad, 7326223) according to the manufacturer’s instructions. Biotinylation of 4SU-residues was carried out in a total volume of 250 μl, containing 10 mM Tris-HCl pH 7.4, 1 mM EDTA and 5 mg MTSEA biotin-XX linker (Biotium, BT90066) for 30 min at room temperature in the dark. RNA was then purified by phenol:chloroform extraction, denatured by 10 min incubation at 65°C and added to 200 μL μMACS Streptavidin MicroBeads (Miltenyi, 130-074-101). RNA was incubated with beads for 15 min at room temperature and beads applied to a μColumn in the magnetic field of a μMACS magnetic separator. Beads were washed twice with pull-out wash buffer (100 mM Tris-HCl, pH 7.4, 10 mM EDTA, 1 M NaCl and 0.1% Tween20). 4SU-RNA was eluted twice by addition of 100 μL 100 mM DTT and RNA cleaned up using the RNeasy MinElute kit (QIAGEN, 74204) using 1050 μL 100% ethanol per 200 μL reaction after addition of 750 μL RLT buffer to precipitate RNA < 200 nt. Amount of 4SU-labbled RNA and size of fragments were confirmed by bioanalyzer prior to library preparation. Libraries for RNA sequencing were prepared using the strand-specific TruSeq total RNA kit (Illumina) using 5 min 65°C fragmentation incubation to anneal primers but to prevent further fragmentation of the samples. The libraries were then sequenced with paired end 100 nt run on the HiSeq 2500 (PE100 run). For DRB/TT-Seq, CRIPSR KO grown for 5 days with and without Dox and treated with 100 μM DRB (5,6-dichloro-1-β-D-ribofuranosylbenzimidazole) (Sigma-Aldrich, D1916) for 3.5 hr. DRB inhibition was released by 3 washes in pre-warmed PBS before addition of fresh media. For the 10 min release, 1 mM 4SU were added directly after the DRB removal and for the following time points 1 mM 4SU were added to the cells 10 min prior to TRIzol addition. RNA was extracted, biotinylated and purified as described above and libraries prepared using the NuGEN ultra low V2 kit (NuGEN). DRB/TT-Seq samples sequenced on a HiSeq 4000 (PE75 run). Reads were aligned against the *Homo sapiens* GRCh38 and *Saccharomyces cerevisiae* sacCer3 genome builds using STAR v2.5.2a (Dobin et al., 2013) with Ensembl release 86 transcript annotations. Resulting genome alignment BAM files were merged at the CRISPR status level, sorted and indexed using Picard v2.1.1 (http://broadinstitute.github.io/picard). The yeast spike-in was used to account for differences in library size between samples. A yeast gene-level counts matrix was generated and passed to DESeq2′s estimateSizeFactors function (Love et al., 2014). An equivalent human count matrix was further analyzed with DESeq2 using the yeast-derived scale factors. PCA analysis was conducted on the rlog transformed counts of the 500 most variable genes.

#### PolyA^+^ mRNA-Seq

RNA from CRIPSR KOs grown for 5 days with and without Dox were harvested using the RNeasy kit (QIAGEN, 74104) according to the manufacture’s instruction including the on-column DNase treatment. 2 μg total RNA was used for oligo(dT) purification and library preparation using the TruSeq HT kit (Illumina). The libraries were sequenced on a HiSeq 4000 (PE100 run). Reads were aligned against GRCh38 and Ensembl release 86 transcript annotations using STAR v2.5.2a (Dobin et al., 2013) via the transcript quantification software RSEM v1.2.31 (Li and Dewey, 2011). Resulting genome alignment BAM files were merged at the CRISPR status level, sorted and indexed using Picard v2.1.1 (http://broadinstitute.github.io/picard). For all single gene examples, sequencing data from cell lines with same genotype were merged and tracks scaled to library size.

#### 3′-Seq

RNA from CRIPSR KOs grown for 5 days with and without Dox were harvested using the RNeasy kit (QIAGEN, 74104) according to the manufactures’ instruction including the on-column DNase treatment. 3′-Seq libraries were prepared as previously described (Hoffman et al., 2016) and sequenced on a HiSeq4000 (SE75 run). Reads were trimmed using Cutadapt v1.9.1 (Martin, 2011) to remove 5bp 5′ anchored barcodes and then aligned against the *Homo sapiens* GRCh38 genome build using Bowtie2 v2.2.9 (Langmead and Salzberg, 2012). Resulting genome alignment BAM files were merged, sorted and indexed using Picard v2.1.1 (http://broadinstitute.github.io/picard).

#### mNET-Seq

mNET-Seq were carried out as described previously (Nojima et al., 2015). Briefly, CRISPR KOs were grown for 5 days with and without Dox prior to harvest. RNAPII was immunoprecipitated using an antibody recognizing all forms of RNAPII (MABI0601, MBL International). Small RNA libraries were prepared using NEBNext Multiplex Small RNA Library Prep Set for Illumina (NEB, E7300S) and sequenced on a HiSeq4000 (Illumina) (PE75 run). Data processing was adapted from Nojima et al., 2015. Briefly, reads were adaptor trimmed using TrimGalore v0.4.4 (Martin, 2011) with the following settings: “–paired–quality 20 -e 0.05 -a AGATCGGAAGAGCACACGTCTGAACTCCAGTCAC -a2 GATCGTCGGACTGTAGAACTCTGAAC–length 10.” Reads < 10bp in length were discarded. HISAT2 v2.0.4 was used to align remaining paired reads against the GRCh38 genome build in a strand-specific manner (Kim et al., 2015). SAMtools was used to remove multi-mapping reads, duplicate reads and those not mapping in proper pairs. The last base incorporated by the polymerase was isolated as the 5′ end of the second mate read and given the strand information of the first mate using the python script “get_SNR_bam.py” (https://github.com/tomasgomes/mNET_snr), resulting in “SNR” level BAM files.

#### PAR-CLIP

Parental Flp-In T-REx HEK293 cells or Flp-In T-REx HEK293 stably expressing Dox-inducible FLAG-SCAF4 or FLAG-SCAF8 were incubated overnight with 1 μg/mL Dox and 100 μM 4SU (Glentham Life Sciences, GN6085). The following day an additional pulse of 500 μM 4SU was added for 2 hr. Cell were UV-crosslinked in a Stratalinker 2400 equipped with 365 nm light bulbs at 2000 J/m^2^, snap frozen in liquid nitrogen and stored at −80°C. Cells were then fractionated into soluble (cytoplasmic and nucleoplasmic) and chromatin fractions and used for FLAG immunoprecipitations as described for ‘FLAG-SCAF4 and FLAG-SCAF8 Immuno-precipitations’ using limited RNaseI digestion (1 U/mL, Thermo Fisher Scientific, AM2294) and TURBO DNase (10 U/mLThermo Fisher Scientific, AM2238) for 2 min at 37°C, followed by 5 min incubation on ice instead of benzonase treatment for chromatin extraction of RNA-protein complexes. Both soluble and chromatin enriched extracts were additionally treated with 30 U/mL RNaseI (Thermo Fisher Scientific, AM2294) for 3 min at 37°C prior to FLAG-IPs which were carried out a described for ‘FLAG-SCAF4 and FLAG-SCAF8 Immunoprecipitations’. SCAF4 and SCAF8 protein-RNA complexes were FLAG peptide eluted. 10% of samples were used for silver stain to verify IP and the remaining 90% treated with 4 mg/mL ProtinaseK (Sigma-Aldrich, 3115887001) with the addition of 2% SDS to the FLAG elution buffer for 30 min at 50°C. Fragmented RNA was extracted using the miRNeasy kit (QIAGEN, 217004) from ProtinaseK treated SCAF4 and SCAF8 protein-RNA complexes and 1% input samples (including on-column DNase treatment), analyzed on bioanalyzer to determine the size range and amount of co-purified RNA, end-repaired using T4 PNK (NEB, M0201S), purified using AMPureXP beads (Beckman Coulter, A63881) and used for small RNA libraries preparation using NEBNext Multiplex Small RNA Library Prep Set for Illumina (NEB, E7300S and E7580S). Final cDNA libraries were gel purified and size selected for inserts between 20-80 nt, which were confirmed by bioanalyzer prior to sequencing. Libraries were sequenced on HiSeq4000 (SE100 run for replicate 1 and SE75 run for replicate 2-3).

### Quantification and Statistical Analysis

#### BigWig files

bigWig files were generated by converting BAM files merged by CRISPR status to bedGraph format using BEDtools’ genomeCoverageBed function (Quinlan and Hall, 2010). Where applicable, yeast scale factors were applied to normalize for differences in library size, otherwise scale factors derived from the human gene count data generated by DESeq2′s estimateSizeFactors function were used. bedGraph files were in turn converted to bigWig format using the bedGraphToBigWig function from the KentTools package (Kent et al., 2010).

#### RNA-Seq alternative isoform analysis

Isoform changes from mRNA-Seq data were computed using the MISO algorithm (Katz et al., 2010). Significant isoform changes were selected as events with a bayes factor ≥ 10 & dPSI ≥ +/−0.3 in KO cells compared to WT cells.

#### Polyadenylation site usage

A set of high-confidence cleavage sites were computed from 3′-Seq data by merging reads from all 3′-Seq samples (regardless of KO status). Reads not overlapping with any annotated Ensembl exon in a strand-specific manner or any with a mapping quality < 10 were discarded. Remaining reads mapping to forward strand exons were filtered to include those with 3′ soft-clipped regions containing an “AA” at their 3′ terminus or “AAA” in any portion of the soft-clipped region. “TT” and “TTT” were used as equivalent soft-clipped region filters at the 5′ end of reads mapping to reverse strand exons. The filtered soft-clipped reads were used to create a read-depth coverage tracks and isolate islands that were at least 10 bp deep in a strand-specific manner. This resulted in 40,215 cleavage sites. A high-confidence cleavage set was generated from cleavage sites associated with a single gene (39,256), that map to either the terminal exon (32,235) or 3′UTR (26,883) of that gene, that have a merged read count of > 300 (28,106), contain at least 1 canonical or non-canonical polyA site (33,866) and represent at least 5% of all merged-reads belonging to the total exonic portions of the gene (20,723). This resulted in 17,835 high confidence cleavage sites. The relative expression difference (RED) scores for genes harboring ≥ 2 high-confidence cleavage sites were calculated using the 3′-Seq read count in the ± 50bp interval surrounding the midpoint of each cleavage site. Cleavage sites mapping to more than one gene were not considered. The usage of cleavage sites was normalized to the usage of the 3′most cleavage site (longest isoform) and taken relative to the WT cells using a previously described method for assessing relative expression differences (RED) from 3′Seq data (Li et al., 2015). RED = log2(short#/long#)_KO_ – log2(short#/long#)_WT_. Thus, a positive RED score indicates an enrichment of the short isoform in the KO relative to the WT.

Genomic distributions of high confidence sites were generated using a set of exon/intron/utr/gene/intergenic definitions based on a single representative Ensembl transcript per protein-coding gene. Priority was given to transcripts with i) a high confidence “Transcript Support Level,” ii) that terminated furthest from the TSS or iii) had the largest exonic footprint.

Motif enrichment analysis of cleavage sites (+/− 50 bp from each midpoint) was conducted using MEME v4.11.12 (Bailey et al., 2009) (meme-chip) against a background of unique Ensembl TSS sites ± 50bp in a strand-aware manner.

#### Nascent RNA-Seq profiles

ngs.plot software was used to generate read coverage profiles over functional genomic regions using the mate1 reads only (Shen et al., 2014). The default ngs.plot Ensembl database definitions were used for TSS, TES and genebody regions. Ensembl gene biotype annotation was used to take slices of the data for all, protein-coding, protein-coding ≥ 120 kb in length, lincRNA, snRNA and snoRNA genes.

#### Readthrough analysis

The transcriptional readthrough analysis was restricted to protein encoding genes with a read count > 30 in the terminal exon (3′most annotated exon including the 3′UTR region) for all four CRISPR groups (WT, *SCAF4* KO, *SCAF8* KO or dKO) and a read count > 10 for the 50 kb downstream of the TSS in all CRISPR groups. Transcriptional readthrough was measured as TT-Seq reads mapping 50 kb downstream of the most distal annotated TES relative to the read count in the terminal exon (including 3′UTR). For ratios relative to WT an additional restriction of a terminal exon read count > 50 and downstream of TES region read count > 50 for each CRISPR group was applied. The 1000 genes with the highest post TES/terminal exon variation passing the basic expression filter was used as a top 1000 set of readthrough genes. A Pearson correlation analysis was conducted using just the 1000 genes with the largest coefficient of variation across all sample level run-through ratios. ngs.plot software was used to generate strand-specific read coverage profiles from the top run-through genes calculated from data merged by CRISPR status (Shen et al., 2014).

#### Nascent transcription across proximal and distal terminal exons

Terminal exons from all multi-transcript protein-coding genes (Ensembl annotation) from high-confidence transcripts (with a support level of 1) were considered. If the terminal exon was < 500 bp it was extended to 500 bp from the center. Strand-specific TT-Seq signal was quantified for all and genes with a terminal exon that failed to show an area of abundance ≥ 1000 in both WT and dKO samples were discarded. Only the remaining most proximal and most distal terminal exon combination was selected per gene for further analysis. This gene list was further filtered to remove genes in which either the proximal or distal exon overlapped a neighboring gene, leaving 6434 genes. Out of these 340 had a significant ALE events as detected by MISO in the WT versus dKO comparison (SCAF4 and SCAF8 regulated). To test whether nascent transcription across the most proximal and most distal terminal exons was affected in those genes we calculated log2 ratios (dKO distal/proximal) / (WT distal/proximal) of strand-specific nascent transcription for the defined terminal exons. As a control 10,000 random sets of genes (n = 340) were selected from the 6094 non-regulated genes (genes without any significant ALE event).

#### Intronic pA sites usage

Coordinates for IpA events were obtained from Singh et al. (2018). To remove IpA sites not expressed in our cell line we disregarded IpA events with a total RPKM < 50 across all samples from our 3′-Seq data in a window of 100 nt around the IpA site. Differentially used IpA sites were computed by counting the number of normalized reads in a 100 nt window around the IpA relative to WT cells.

#### DRB/TT-Seq analysis

Base-pair coverage of the TSS region −2kb:120kb of Ensembl protein coding genes 60-300kb in width (n = 4,869) were calculated from the BAM files and converted to read-counts per million (RPM). Gene meta-profiles were created by taking a trimmed mean (0.01) across each position and fitting a smoothing.spline using R’s smooth.spline function (means.spar = 0.9) to the averaged data.

#### Wave peak calling

Wave peaks were calculated from the positions at which the splines reached their maxima, with the requisite that the wave advanced with increasing DRB release time. Elongation rates were calculated based on a linear regression assuming that the wave peak position at time 0 is at the TSS. Single gene wave peaks were calculated as above using the smooth.spline function with additional filters requiring that (1) genes were expressed at an RPM > 100 across all samples, (2) the wave peak for the 10 min time-point was called after the first 2 kb across all cell lines, (3) genes with any missing values were disregarded (4), genes with duplicate wave peak positions for successive time-points were removed and (5) the wave peak progressed from the 10, 20 and 30 min time points across all cell lines. This left 189 genes for single gene elongation rate calculations. Since the 40 min wave peak sometimes extended beyond the annotated individual gene, only the 10, 20 and 30 min wave peak positions were used to calculate single gene elongation rates.

#### PAR-CLIP analysis of SCAF4 and SCAF8 binding sites

Reads were adaptor trimmed using the CutAdapt (Martin, 2011) wrapper TrimGalore with the following settings:

“–quality 20 –e 0.1 –a AGATCGGAAGAGCACACGTCTGAACTCCAGTCAC –length 10.” Trimmed reads were aligned against the *Homo sapiens* GRCh38 genome build using Bowtie2 v2.2.9 (Langmead and Salzberg, 2012) with the following settings: “-S –n 2 –m 100 –k 1 –1 10 –chunkmbs –best–strata.” Resulting genome alignment BAM files had duplicated reads removed, sorted and indexed using Picard v2.1.1 (http://broadinstitute.github.io/picard). Reads from the chromatin and soluble fractions for each replicate were merged into a single BAM file. To access the RNA-protein crosslink T→C signature, all nucleotide conversions were counted in uniquely mapping reads. PAR-CLIP libraries were analyzed using the PARalyzer pipeline (Corcoran et al., 2011). Reads both from soluble and chromatin enriched fractions were combined prior to cluster calling. Clusters were required to consist of > 10 reads (non-duplicate reads) and containing ≥ 8 TC transitions in ≥ 8 positions. As an addition step to remove background clusters (normally this control is not done for CLIP experiments), clusters overlapping with more than one cluster from the input samples were removed and genes containing ≥ 1 input cluster / 10 kb was disregarded leaving us with a highly stringent set of SCAF4 and SCAF8 binding clusters. Consensus sets of SCAF4 and SCAF8 RNA-binding clusters were generated from clusters overlapping in 2 out of 3 biological replicates. A consensus set of target genes were defined for both SCAF4 and SCAF8 as genes harboring ≥ 1 consensus cluster(s). This resulted in 14,783 SCAF4 and 8,534 SCAF8 RNA-binding clusters in 3,082 and 1,924 target genes, respectively. As a confirmation of the selection criteria this resulted in only 125 background clusters (corresponding to 6 genes) from our control CLIP experiment (pull-out from parental cell not expressing a FLAG-tagged protein). The similarity between SCAF4 and SCAF8 binding was assessed by computation of Jaccard correlation coefficients for each pair of samples. The Jaccard similarity coefficient runs from 0-1 and was calculated as (intersect/union) with a value of 1 indicating that the two sets are identical. Due to the high similarity between the individual SCAF4 and SCAF8 replicates, we further included a pooled set of SCAF4 *and* SCAF8 RNA-binding clusters taking into account both SCAF4 and SCAF8 consensus clusters in addition to cluster with overlapping binding evidence from either SCAF4 or SCAF8 by considering all clusters found ≥ 2 out of 6 SCAF4 *or* SCAF8 samples. The genomic distribution of RNA-binding clusters was computed using the cluster midpoint. Transcripts were defined as the longest isoform including 2 kb upstream of the TSS and 2 kb downstream of the TES. For localization of binding clusters within transcripts an upstream region was defined as 2 kb upstream of the TSS and a downstream was defined as 2 kb downstream of the 3′ most annotated TES.

Metagene and exon-intron/intron-exon centered cluster coverage plots were computed using the pooled set of SCAF4 and SCAF8 clusters as well as the SCAF4 and SCAF8 consensus cluster sets. Meta-transcriptome profiles of SCAF binding clusters were created by concatenating exonic cluster coverage per transcript, fitting a smoothing spline to estimate coverage over 100 equally sized bins and taking a trimmed mean (0.01) of average depth per bin over all transcripts.

#### RNAPII mNET-Seq analysis

Meta-gene profiles of the SNR-level mNET-seq data over protein-coding genes were created using ngs.plot software (Shen et al., 2014). The exon/intron and intron/exon plots were created from the SNR-level data using sets of intervals ± 400 bp either side of the junctions extracted from the Ensembl annotation. A single representative transcript was selected per protein-coding gene. Priority was given to transcripts with i) a high confidence “Transcript Support Level,” ii) that terminated furthest from the TSS or iii) had the largest exonic footprint. Coverage was calculated at a bp-level. A trimmed mean (0.01) of the coverage across all available sites was calculated for each bp. Profiles were scaled to library size. RNAPII Ser2P and Ser5P mNET-Seq data from Nojima et al. 2015 were downloaded from GEO (GSE60358).

#### Definition of SCAF4 and SCAF8 regulated genes

SCAF4 and SCAF8 regulated genes were grouped into 3 categories: *i.* genes with early polyA site selection and termination events identified from *both* mRNA-Seq data and 3′-Seq; *ii*. genes with polyA and termination site change identified from mRNA-Seq alone; and *iii*. genes with early polyA and termination identified from 3′-Seq data alone. Class *i* are comprised of genes with evidence of early polyA site selection and termination events from both mRNA-Seq data as well as 3′Seq and include genes containing a significant MISO event and 3′Seq changes toward shorter isoform in dKOs *or* genes with upregulated IpA sites in dKOs supported by mRNA-Seq data *or* genes with both a 3′Seq supported upregulated IpA and a MISO ALE events in dKOs. Class *ii* consists of genes with evidence of polyA and termination site change from mRNA-Seq alone and include genes with ALE events in dKOs but without a positive RED score or without ≥ 2 high-confidence cleavage sites from the 3′Seq *or* IpA events exclusively supported by mRNA-Seq data. Class *iii* includes genes with increased IpA usage in dKOs but without accompanied increased mRNA-Seq signal. Fisher exact tests were used to test the significance of the overlap between the various SCAF regulated gene classes with SCAF pooled target genes as defined by PAR-CLIP.

#### Motif analysis

Motif analysis were performed using the MEME-ChIP algorithm (Machanick and Bailey, 2011) on clusters resized to a width of 100 bp around their midpoint. A background was defined as clusters seen in at least 2 of the 9 input samples.

#### Distance between polyA sites and splice junctions

The proximal sites of MISO ALE events or IpA sites with increased mRNA-Seq signal in *SCAF4 SCAF8* dKOs were analyzed to see if there was a bias in their position relative to the upstream exon/intron junction. A single longest transcript was selected per protein-coding gene. Only events where the proximal site mapped to the intron of the representative transcript were considered. Distances were calculated using the 3′ most base the proximal site. The observed distances were defined as the median width of the intron to which the proximal site maps. A binomial test was used to assess the significance of the observed sites residing in the 5′ half of introns.

#### RNAPII speed mutants

mRNA-Seq data from slow RNAPII (R749H), WT RNAPII and fast RNAPII (E1126G) from Fong et al. (2014) were downloaded from GEO: GSE63375.

### Data and Software Availability

All TT-Seq, DRB/TT-Seq, mRNA-Seq, 3′-Seq, mNET-Seq and PAR-CLIP data used in this study are available under GEO: GSE121826.

## Supplemental Information

Table S1. Label-free Protein Quantification of FLAG-SCAF4 and FLAG-SCAF8 IPs, Related to Figure 2

Table S2. Genes Regulated in a SCAF4 or SCAF8-Dependent Manner, Related to Figures 3, 5, and 6

Table S3. List of Reagents, Plasmids, and SRA Samples that Are Subject of this Study, Related to STAR Methods
